# Hand hygiene among cleaning staff in acute care hospitals: a scoping review

**DOI:** 10.3389/fpubh.2026.1800229

**Published:** 2026-07-16

**Authors:** Anna-Maria Rager, Viviane Scherenberg

**Affiliations:** 1 Faculty of Human and Health Sciences (FB 11), University of Bremen, Bremen, Germany; 2 Public Health and Environmental Health, APOLLON University of Applied Sciences, Bremen, Germany

**Keywords:** cleaning staff, hand hygiene, hand hygiene compliance, hand hygiene interventions, hospital cleaning, hospitals, housekeeping, infection control

## Abstract

**Objectives:**

The aim of this study was to examine attitudes and behaviors related to hand hygiene among hospital cleaning staff and to identify promoting and inhibiting factors influencing hand hygiene behavior. In addition, existing worldwide target group-specific interventions for this occupational group were mapped.

**Methods:**

A comprehensive literature search was conducted for publications from 2009 to 2025 in MEDLINE (via PubMed^®^), the Cochrane Library, CINAHL (via EBSCO), Embase, and Scopus. Additional research was carried out in WHO IRIS, BASE, and Science.gov, and internationally applicable guidelines were identified using the TRIP Database. Included were studies from worldwide somatic acute care hospitals examining hand hygiene compliance, knowledge, attitudes, practices, influencing factors, or interventions among cleaning staff. Texts limited to other hygiene domains, airborne infections, non-acute-care settings, or study populations consisting of mixed occupational groups and not limited to cleaning staff exclusively were excluded. Literature screening was performed in two stages (title/abstract and full-text screening), and data extraction was conducted by one reviewer using a standardized extraction table.

**Results:**

In total, 33 sources met the inclusion criteria, comprising five guidelines, 25 full-text studies, and three conference abstracts, which formed the primary basis of this review. The conference abstracts identified during the research for which corresponding full texts were not provided were documented for the sake of completeness, but not included in the main analysis and qualitative synthesis, as relevant information may not be available due to the lack of full texts. The base of evidence was heterogeneous and predominantly descriptive, addressing knowledge, attitudes, practices, as well as promoting and inhibiting factors. Identified interventions related to infection prevention and control and hand hygiene among cleaning staff were based on the provision of target group-oriented training materials, simulation-based training, and multimodal approaches and training programs.

**Conclusion:**

The findings indicate overall limited and predominantly descriptive evidence regarding hand hygiene among hospital cleaning staff. This underscores the need for methodologically diverse research as well as the development of target group–specific interventions and guidelines to strengthen infection prevention, particularly hand hygiene, in acute care hospital settings.

## 1 Introduction

The World Health Organization (WHO) defines the term “patient safety” in its 2009 “WHO Patient Safety Curriculum Guide for Medical Schools” as “[...] a concern that affects everyone – healthcare professionals, cleaning and catering staff, managers, bureaucrats, consumers, and politicians” (1). This conception of patient safety also applies to the prevention of healthcare-associated infections (HAIs). Basic hygiene is a fundamental component of HAIs prevention and represents a multifactorial concept in which several interrelated measures complement one another. These include hand hygiene, surface hygiene, barrier measures, medical device reprocessing, waste disposal, linen handling, bed hygiene, tableware handling, education of patients, and visitors regarding personal hygiene measures as well as isolation of patients at increased risk of transmission (1, 2). When considering basic hygiene from the perspective of individual occupational groups, it becomes apparent that cleaning staff, the third largest group of employees in hospitals, carry out a substantial proportion of these measures as part of their daily work. Hand hygiene is considered the most important measure for reducing nosocomial infections and reducing the transmission of antibiotic-resistant pathogens. The umbrella term *hand hygiene* encompasses both handwashing with soap and water as well as hand disinfection, frequently in the form of alcohol-based hand sanitizers. The distinction between handwashing and hand disinfection is particularly relevant with regard to hand hygiene compliance (HHC), as these two measures differ in terms of their indications within a clinical context (1). Regarding hand hygiene, the “Five Moments of Hand Hygiene” concept (WHO) must be implemented by all professional groups with direct or indirect contact with patients (2). The interventions studied by Pittet et al. (3) show a decrease in the incidence of nosocomial infections from 16.9% to 9.9% by increasing staffs' adherence to HHC from 47.6% to 66.2%. Nevertheless, non-compliance with adequate hand hygiene continues to be a significant issue in healthcare facilities among all professional groups, for instance, physicians, nurses, cleaning staff, etc. (1–4). According to the study by He et al. (5), the “Five Moments of Hand Hygiene” approach (WHO) is assumed to be less consistently adhered to by cleaning staff. This is especially true because this approach is difficult to implement into the daily work of cleaning staff. Studies in recent years have contributed to a more comprehensive understanding of HHC (4–6). In 2009, the WHO Guidelines for Hand Hygiene in Healthcare were published as part of the first WHO global patient safety initiative (2). They provide a comprehensive and evidence-based overview and therefore represent a relevant basis for improving hand hygiene behavior in healthcare facilities. Departing from the base of these guidelines, the development of target group-oriented interventions considering the work processes of all healthcare stakeholders is an important and promising approach for optimizing HHC (4–6). Previous approaches to improving HHC have mostly focused on healthcare professionals. Studies and interventions for professional groups without direct patient contact, especially cleaning staff, are rare (7), and further research in this area has been called for (5). A preliminary search of MEDLINE (via PubMed^®^), the Cochrane Library, International Prospective Register of Systematic Reviews (PROSPERO), and Open Science Framework (OSF) did not reveal any current or ongoing systematic or scoping reviews nor research syntheses on this topic. Consequently, this review attempts to offer insight into this untreated intersection. In addition, it will provide a comprehensive overview on prevailing research regarding the current state of research on hand hygiene among cleaning staff in acute care hospitals worldwide. The foci consist of identifying knowledge, attitudes, and practices (KAP) as well as factors that promote and inhibit hand hygiene behavior, and identifying already established target group-specific interventions for cleaning staff in hospitals. The focus on KAP as well as on factors that facilitate or hinder hand hygiene arises from the observation that relevant insights regarding this target group already exist but remain scattered across individual studies and have not yet been systematically synthesized. Consolidating this evidence will support a clearer and more comprehensive understanding of the needs, challenges, and existing capacities of cleaning staff in healthcare settings.

The research question to be answered is therefore: What is the current level of knowledge regarding hand hygiene of cleaning staff in acute care hospitals for human healthcare with regard to KAP, promoting and inhibiting factors, and intervention possibilities?

## 2 Methods

The scoping review was conducted in accordance with the JBI methodology for scoping reviews using the JBI Manual for Evidence Synthesis (2024) for Scoping Reviews (8).

The checklist “Preferred Reporting Items for Systematic reviews and Meta-Analyses extension for Scoping Reviews” (PRISMA-ScR) was used to verify the reporting standard of the scoping review by Tricco et al. (9). This scoping review was registered via the Open Science Framework (OSF, 10 May 2025, registration DOI: https://doi.org/10.17605/OSF.IO/ZCK34).

DeepL Translator (free version), Google Translate and Chat GPT (paid version, version 5.1) were used to translate identified sources from French, Turkish, and Chinese to German. When possible, ambiguous text passages were translated using both DeepL Translator and Google Translate, and then compared. All translations were manually checked by the authors, assuring mutual quality control and by means of back-translation and comparison with the original texts.

### 2.1 Search strategy

The inclusion and exclusion criteria were formulated based on the population, concept, context (PCC) approach, and supplemented by the aspect of source types. The inclusion and exclusion criteria used are listed in abbreviated form below in the text; the detailed version is provided in Appendix 1. The search for literature published between 2009 and 2025 (2009: publication of the WHO guidelines on hand hygiene in healthcare (2)) was conducted between May and June 2025. There were no language restrictions (10).

A three-stage search strategy was used to compile the scoping review: Stage 1: A limited search was conducted in at least one database relevant to the topic, in this case MEDLINE (via PubMed^®^), in order to identify articles relevant to the topic. The titles and abstracts of the articles identified were analyzed for index terms and keywords. An information scientist from the Leibniz Institute for Prevention Research and Epidemiology – BIPS was involved in the development, and particularly the refinement of the search strategy (see Appendix 2.1). Stage 2: A comprehensive search strategy was developed based on these keywords and index terms. The search strategy, including all identified keywords and index terms, was customized for each database and/or information source included (using the Polyglot Search Translator, TERA Tools). Separate searches were conducted in MEDLINE (via PubMed^®^), Cochrane Library, CINAHL Database (via EBSCO), Embase, and Scopus. The final search strings developed for MEDLINE (via PubMed^®^), based on the PCC scheme, are listed in Appendix 2.1.

The individual search strands (1, 2, 3), supplemented by the relevant period, were connected with the Boolean operator AND. The final search strings for all databases are provided in Appendix 2.2 for reproduction.

The WHO Institutional Repository for Information Sharing (IRIS), Bielefeld Academic Search Engine (BASE) and Science.gov databases were used to research gray literature. The TRIP Medical Database was used to identify internationally applicable guidelines (see Appendix 2.3). Consensus, an AI-powered academic search engine, was used as an additional research and comparison tool (see Appendix 2.4). Stage 3: The reference lists of all included sources were checked for further relevant studies. These results were documented systematically. Literature without direct access options was obtained via interlibrary loan and author requests.

### 2.2 Inclusion and exclusion criteria

Following the PCC framework, inclusion and exclusion criteria were formulated and considered in the systematic literature search and selection. The framework was further supplemented by the criterion “source types.”

#### 2.2.1 Population

We included cleaning staff and other occupational groups without direct patient contact, such as cleaners, and building technicians in acute care hospitals worldwide. Mixed populations comprising cleaning staff, nursing staff, and physicians were excluded when separate results for cleaning staff were not reported. In many of these studies, cleaning staff represented only a small proportion of the overall sample, limiting the relevance and interpretability of the findings for the target group and preventing a sufficiently target-group-specific analysis. This approach was intended to ensure greater methodological consistency and target-group specificity. Studies focusing exclusively on occupational groups with direct patient contact, such as nurses and physicians, were also excluded.

#### 2.2.2 Concept

We considered texts assessing the current situation regarding compliance and KAP, as well as factors that promote or hinder hand hygiene. The hand hygiene measures considered were hygienic hand disinfection, handwashing, and the combination of hygienic hand disinfection and handwashing. Studies examining infection prevention and control (IPC) measures more broadly also met the inclusion criteria, provided that they explicitly addressed hand hygiene. Possible interventions included reminder systems, training and continuing education programs, electronic systems, and multifactorial approaches. Studies focusing on the current situation with an emphasis on skin care and protection as well as surface and textile hygiene, without a clear link to hand hygiene, were excluded. Furthermore, data collection and interventions related to infectious diseases mainly transmitted by airborne pathogens were not considered.

#### 2.2.3 Context

We included studies on somatic acute care hospitals worldwide. Studies focusing on veterinary facilities, psychiatric facilities, and non-medical facilities were excluded.

#### 2.2.4 Types of sources

In terms of the relevant study designs, this review included observational studies, experimental, and quasi-experimental as well as qualitative and interventional study designs in addition to various types of systematic reviews with and without meta-analyses. Additional literature such as expert interviews, reports from professional societies, and national and international guidelines, was included in the analysis. There were no exclusion criteria regarding source types.

### 2.3 Source of evidence selection

The freely available literature management software Zotero (Digital Scolar, 6.0.36, 2024, Vienna, VA, USA) was used to record and manage the identified literature. The identified literature was uploaded to both Zotero and Rayyan, an AI-powered platform for managing systematic reviews. Deduplication was performed and compared using both programs.

The screening process for the identified literature was carried out in two steps using the Rayyan platform. The first step involved screening the titles and abstracts, which were reviewed by two independent reviewers based on the predefined inclusion criteria for the review (see Appendix 1). Interrater agreement in the title and abstract screening was 95.9% (725/756) with 31 discrepancies. Interrater reliability for the title and abstract screening was assessed using Cohen's kappa. The resulting kappa coefficient was κ = 0.78, indicating substantial agreement (0.61–0.80) between reviewers. If a title and abstract were deemed relevant, the second step involved a full-text review (8). Due to resource constraints, approximately 25% of the randomly selected full texts were screened jointly by two reviewers. The remaining texts were screened by one reviewer, with a second and third reviewer consulted in cases of uncertainty. The literature excluded in step 2, along with the reasons for exclusion, is listed in a separate table, which is part of the Supplementary material. In case of uncertainty regarding the inclusion and exclusion criteria of individual literature, an additional reviewer could be consulted.

During the research work, conference and poster abstracts without accompanying full text were also identified. These are also listed in the Supplementary material in the detailed extraction table, but not included in the main analysis and qualitative synthesis, as relevant information may not have been available due to the lack of full text and therefore could not be considered.

**Figure 1:**
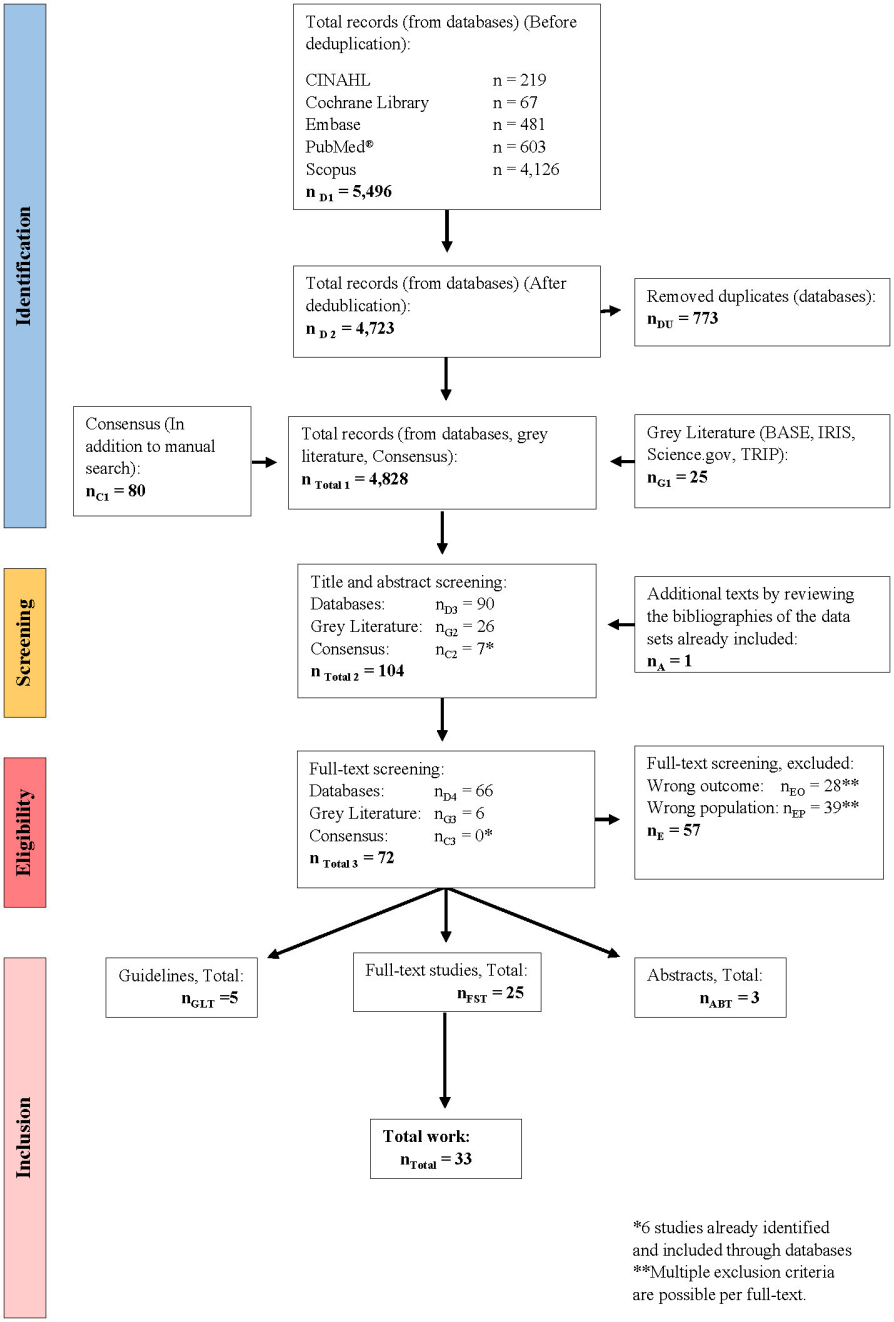
PRISMA flowchart [9]. n_A_, Additional identified texts; n_ABT_, Abstracts, total; BASE, Bielefeld Academic Search Engine; n_C_, Text, from Consensus (AI); CINAHL, Cumulative Index to Nursing and Allied Health Literature; n_D_, Texts from databases (CINAHL, Cochrane Library, Embase, PubMed^®^, Scopus); n_DU_, Duplicate; n_E_, Texts, excluded (based on various grounds for exclusion); n_EO_, Texts, excluded due to unsuitable outcomes; n_EP_, Texts, excluded due to unsuitable target group/population; n_FST_, Full-text studies, total; n_G_, Texts, gray literature (BASE, IRIS, Science.gov, TRIP); n_GLT_, Guidelines, total; IRIS, Institutonal Repository for Information Sharing; TRIP, Original meaning of the abbreviation (no longer current): Turning Research Into Practice; WHO, World Health Organization.

The results of the search and the process of inclusion and exclusion of literature are presented in a PRISMA flow diagram (41) (see Figure 1).

### 2.4 Data extraction

The identified texts were divided into three self-defined categories (see Appendix 3.1). A pilot screening was conducted using randomly selected texts from the included studies to test the suitability of the self-developed data collection form, consisting of an Excel spreadsheet (see Appendix 3.2).

### 2.5 Data analysis and presentation

The data were presented in narrative and tabular form, considering the relevant thematic priorities. Flowcharts, diagrams, and graphs were used to additionally illustrate relevant relationships. Based on the recommendations of the JBI Manual for Evidence Synthesis (2024), the quality of the evidence was not assessed during the preparation of the scoping review (8). To ensure transparency and quality of reporting, only studies with full texts available were included in the manuscript. For several identified conference and poster abstracts, corresponding full texts could not be retrieved, resulting in insufficient information for an adequate evaluation of these studies. Therefore, contents of these abstracts are listed under “Supplementary material.” The Supplementary material primarily provides additional details as well as expanded presentations of results that support the synthesized findings presented in the main manuscript. Where possible, findings specifically related to hand hygiene were distinguished from broader IPC-related findings during data synthesis and presentation, particularly regarding promoting and inhibiting factors.

## 3 Results

### 3.1 General information

The Searches in MEDLINE (via PubMed^®^), the Cochrane Library, CINAHL (via EBSCO), Embase, and Scopus, as well as supplementary searches in WHO IRIS, BASE, Science.gov, the TRIP Database, and Consensus, yielded a total of 4,828 results. During the screening of titles and abstracts, 104 sources were deemed suitable. In the subsequent full-text screening, 72 sources were found to be suitable. Of the 72 identified sources, a final total of 33 sources were included across the categories of guidelines, full-text studies, and abstracts (conference and poster abstracts without associated full text), including five guidelines, 25 full-text studies, and three abstracts (40, 42, 43). The PRISMA flow diagram (Figure 1) illustrates the screening and selection process in greater detail (41).

### 3.2 Guidelines

A total of five guidelines met the inclusion criteria: four with international focus and one with a national focus on Singapore. Three of the four internationally applicable guidelines (11–13) originate from the WHO in Geneva and one from the WHO regional office in Europe (14). The guideline focusing on Singapore originates from the Ministry of Health Singapore [National Infection Prevention and Control (NIPC) Committee] (15). The included texts are an aide-mémoire (14), a training package consisting of three thematically successive parts (11–13), and a national standard (15) on the topics of cleaning and disinfection. None of the guidelines focus exclusively on hand hygiene for cleaning staff, but rather on IPC among cleaning staff in healthcare facilities in general, covering topics such as general education and training, occupational health and safety, personal hygiene, cleaning equipment, the use of personal protective equipment (PPE), surface cleaning, disinfection, waste as well as laundry management. Hand hygiene measures are considered part of an integrated approach. Table 1 lists information on publishing institutions (year of publication), continent, country (scope), document type, setting, population, thematic focus, and key findings.

**Table 1:** Overview guidelines.

References	Continent, country (Scope)	Document type	Setting, population	Thematic focus	Key findings
World Health Organization (11)	Worldwide, low-and middle-income countries	Guideline, part of a training package (Part I of III), Trainer's guide	Healthcare facilities in low-and-middle-income countries, people who perform environmental cleaning in healthcare facilities (cleaning staff), especially their trainers.	Training package on environmental cleaning and IPC, to improve knowledge and skills using a practical educational approach for adults. This section focuses on those responsible for training cleaning staff, as well as teaching and learning methods.	Development of eight structured training modules on hospital cleaning procedures; one module focuses on the role of hand hygiene (Module 3 – Hand hygiene) in interrupting infection chains, covering key moments, correct techniques for handwashing and disinfection, and common errors; includes practical exercises and demonstration materials such as various soap forms, disposable drying materials, and alcohol-based sanitizers; recommends addressing these topics during International Hand Hygiene Day (05.05.) to reinforce awareness and compliance.
World Health Organization (12)	Worldwide, low-and middle-income countries	Guideline, part of a training package (Part II of III), Modules and Resources	Healthcare facilities in low-and-middle-income countries, people who perform environmental cleaning in healthcare facilities (cleaning staff), especially their trainers.	Training package on environmental cleaning and IPC, to improve knowledge and skills using a practical educational approach for adults. This part includes the content aspects (modules and resources) that should be taught by competent trainers.	Development of eight comprehensive training modules on IPC, with multiple sections addressing hand hygiene across different contexts (the main module on hand hygiene is Module 3); content covers infection routes, nosocomial infections, and standard precautions including PPE and surface hygiene; specific modules focus on respiratory and personal hygiene, barriers to hand hygiene such as time constraints, skin irritation, and infrastructure issues, and key moments for hand hygiene during cleaning activities; key hand hygiene moments for cleaning staff as defined in the guideline include: before starting work, before donning gloves (for cleaning tasks and waste handling), before contact with clean laundry, after contact with used laundry (even when gloves are worn), after contact with waste (even when gloves are worn), after contact with body fluids (even when gloves are worn), after touching patient-environment surfaces (even when gloves are worn), after handling cleaning agents (even when gloves are worn), and after contact with contaminated cleaning equipment (even when gloves are worn); additional modules link hand hygiene to environmental cleaning, waste and laundry management, and PPE handling; includes visual materials and checklists to support training implementation and performance evaluation.
World Health Organization (13) World Health Organization Regional Office for Europe (14)	Worldwide, low-and middle-income countries Worldwide, in particular Europe	Guideline, part of a training package (Part III of III), evaluation methodology guide [Not a stand-alone intervention, a guide for informed evaluation of the WHO training package (Part I, Part II and Part III)] Aide-mémoire, including checklist	Healthcare facilities in low-and-middle-income countries, people who perform environmental cleaning in healthcare facilities (cleaning staff), especially their trainers. Healthcare facilities, environmental cleaning staff	Training package on environmental cleaning and IPC designed to enhance knowledge and skills using a practical educational approach for adults. This part allows for a thorough assessment of the WHO training package (This document is not counted as a separate/additional part). Aide-mémoire, including checklist about cleaning, waste management and linen management in relation to infection prevention.	Presentation of two training and assessment projects (CLEAN Cambodia and CLEAN Frontline) based on the WHO training package; checklists include questions on infection chains, use of protective gloves, and existence of hand hygiene policies, protocols, and materials; assess implementation of hand hygiene practices during cleaning tasks, including before entering and leaving wards and between patient contacts; highlight the importance of accessible hand hygiene stations and supplies; training integrates theoretical and practical components on hand hygiene, PPE, environmental cleaning, waste and linen management, and personal hygiene; identified barriers include limited infrastructure, insufficient water access, inadequate product availability, and low staff knowledge and motivation. Regular review and update of training on PPE, hand hygiene, and cleaning procedures; inclusion of cleaning staff and IPC personnel in training development; audits to cover cleaning, disinfection, and PPE practices.
Ministry of Health Singapore, National Infection Prevention and Control (NIPC) Committee (15)	Asia, Singapore	National standard, including self-assessment checklist.	Acute healthcare facilities, cleaning staff in healthcare facilities	National cleaning standard, as a self-assessment checklist, based on various components of cleaning and environmental hygiene in acute care.	Core standards for human resources management require hospitals to provide structured basic IPC training for cleaning staff; induction sessions must include key topics such as hand hygiene, correct use of PPE, prevention of exposure to blood and body fluids, and sharps safety; emphasize occupational health and safety through provision of adequate protective clothing and gloves when handling microorganisms and chemicals; core elements are reflected in the accompanying checklist for monitoring compliance and training implementation.

HHC, Hand hygiene compliance; IPC, Infection Prevention and Control; NIPC, National Infection Prevention and Control (Comitee); PPE, Personal protective equipment; WHO, World Health Organization.

The included guidelines consistently emphasize the importance of multimodal IPC approaches tailored to the practical realities of cleaning staff's work (11–15). Recommended implementation strategies include structured IPC training programs, regular workplace-based assessments, the integration of hand hygiene into routine cleaning and disinfection procedures, and the correct use of personal protective equipment (PPE) (11–15).

Hand hygiene is addressed in the context of infection chains, relevant hand hygiene moments, handwashing and hand disinfection techniques, common implementation errors, and the availability of necessary hygiene resources (11–15). Limited infrastructure, inadequate access to water and hygiene products, lack of time, skin irritation, insufficient hand hygiene knowledge, and low staff motivation are identified as major barriers to implementation (11–13).

The guidelines further recommend combining theoretical and practical training approaches while considering principles of adult education (11–13). Suggested strategies include practical exercises, visual teaching materials, workplace-based learning, and activities related to Global Hand Hygiene Day (5 May) (11–13). Specific recommendations regarding the adaptation of hand hygiene procedures to routine cleaning workflows were particularly highlighted in one guideline, including hand hygiene when entering and leaving wards, before contact with the patient environment and clean equipment, between patient contacts, and after contact with contaminated surfaces, equipment, or waste (13).

In addition, collaboration between cleaning staff and IPC specialists is repeatedly emphasized, particularly regarding the joint implementation of audits and the planning of IPC measures adapted to local working conditions (11, 13, 14).

### 3.3 Current situation regarding hand hygiene

A total of 16 studies assessing the current situation regarding IPC, particularly hand hygiene, among cleaning staff in healthcare facilities were identified. Fourteen of these studies used a cross-sectional study design (16–28, 31) and two were methodological studies (29, 30). The studies originated in Pakistan, Saudi Arabia, China, Turkey, India, Germany, Southeast Africa (Republic of Malawi), East Africa (Uganda, Ethiopia), Australia, England, and the USA (Pennsylvania). Data collection methods included covert and open observations, interviews, questionnaires, focus group discussions, epidemiological, and microbiological methods. The core information recorded in the included studies can be divided into the following five categories: (1) Sociodemographic data of the target group, (2) Organizational data, e.g., training measures, especially IPC training, attendance, and participation in IPC committees, availability of equipment (hand hygiene, cleaning, protective clothing), vaccinations, (3) KAP regarding IPC in general, especially with regard to hand hygiene, (4) Factors that promote and inhibit infection prevention and control in general, especially hand hygiene, (5) Microbiological and epidemiological data, e.g., prevalence of colonization with Methicillin-resistant *Staphylococcus aureus* (MRSA), *Enterobacteriaceae* spp., Candida albicans, and incidences of MRSA per 100,000 hospital bed days. Two of the 16 studies focus on hand hygiene among cleaning staff (17, 24), while the remaining 14 studies address IPC among cleaning staff in healthcare facilities in general (16, 18–23, 25–31). Topics include general training and further training, cleaning organization, occupational health and safety, personal hygiene, cleaning equipment, the use of PPE, surface cleaning and disinfection, as well as waste and laundry management. Hand hygiene measures are considered part of an integrated approach. Table 2 lists information on author (year of publication), continent, country, study design, data collection, setting, population, sample size (*n*), thematic focus, relevant keywords, outcomes, and relevant key findings.

**Table 2:** Overview of studies on the current situation regarding IPC in general and hand hygiene in particular (as a single factor or in connection with infection prevention and control) among cleaning staff.

References	Continent, country	Study design, data collection	Setting, population, sample size (*n*)	Thematic focus, relevant keywords	Outcomes	Key findings
Ahmed et al. (16)	Asia, Pakistan (Rawalpindi district)	Cross sectional study (Interview)	Hospital, hospital sanitary worker (*n* = 88)	Assessment of awareness and knowledge among sanitary workers in hospitals with regard to infection control (including training interventions, vaccinations, hand hygiene, PPE, handling of infectious waste). Keywords: Sanitary workers, waste disposal, hospital wastes	Medical examinations and consultations before/after starting work, vaccinations, availability, use of PPE, handling of infectious waste, use and handling of cleaning agents and disinfectants, hand hygiene behavior, awareness on IPC	100% of sanitation workers had no medical examination before or during employment; only 4.54% (3.46%−5.25%) received pre-employment training, and none received in-service training; protective glove use reported by 23.86% (*n* = 21), while 76.14% (*n* = 67) did not use gloves, with surgical gloves being the most common type; hand hygiene practices after handling medical waste were insufficient-−39.77% (*n* = 35) washed hands with water, 52.27% (*n* = 46) with soap and water, and only 7.95% (*n* = 7) used antiseptics; knowledge and awareness of infectious diseases were low, with 27.27% (*n* = 24) reporting awareness, highlighting major gaps in training, PPE use, and HHC.
Alqurashi et al. (17)	Asia, Saudi Arabia	Cross-sectional study, incl. laboratory analysis.	Hospital, healthcare cleaning staff (*n* = 96)	The role of cleaning staff in the development and transmission of nosocomial infections. The focus is on investigating colonization with pathogenic fungi and bacteria, in particular MRSA, *Enterobacteriaceae* and Candida species. Keywords: hospital cleaning staff, hospital hygiene, MRSA, Enterobacteriaceae, HAIs/nosocomial infections	Prevalence of colonization with MRSA, *Enterobacteriaceae* or Candida species	Overall colonization rate: MRSA 9.4%, *Enterobacteriaceae* 15.6%; 283 isolates identified-−134 nasal and 149 hand samples (53%); Gram-positive bacteria predominated in hand samples (83.3%), including Staphylococcus aureus (6.3%) and MRSA (3.1%); Gram-negative bacteria accounted for 8.3%, mainly *Enterobacteriaceae* (4.2%) with *Klebsiella* spp. (2.1%); no significant differences in colonization by demographic group, department, hand hygiene method, or shift (*p* > 0.05); significant association between handwashing frequency and MRSA colonization (*p* = 0.047); combined sample analysis showed association between shift work and *Enterobacteriaceae* colonization (*p* = 0.008), higher in day shift workers (23.3% vs. 2.8%); logistic regression indicated significant associations for *Enterobacteriaceae* colonization with night shift work (AOR = 0.017, *p* = 0.015) and hourly handwashing (AOR = 0.024, *p* = 0.028).
Dutta and Mishra (18)	Asia, India (Kolkata, Kalkutta)	Cross-sectional study	Hospital, hospital cleaning staff (*n* = 260)	Assessment of the KAP of hand hygiene among cleaning staff. Keywords: Hand hygiene, housekeeping staff, personal protective equipment, training.	Sociodemographic and subject-specific outcomes and aspects: Training received/not received on hand hygiene, knowledge of infectious diseases that can be prevented by properly implemented hand hygiene measures	26.5% of participants had not received any training on hand hygiene or hygiene topics; despite training, 139 participants showed insufficient knowledge of infectious diseases preventable through hand hygiene; 60.4% practiced good hand hygiene, 37.3% had adequate knowledge, and about 80% demonstrated positive attitudes; 30.4% wore jewelry, including religious items; 60.8% of those with satisfactory knowledge and 58.5% with negative attitudes still demonstrated good hygiene practices; binary logistic regression revealed significant associations: working in pediatrics and obstetrics positively associated with sufficient knowledge (OR = 4.6, *p* = 0.003); male gender negatively associated with positive attitude (OR = 0.35, *p* = 0.025); working in surgery positively associated with positive attitude (OR = 3.7, *p* = 0.022); professional experience >5 years negatively associated with good practice (OR = 0.44, *p* = 0.048); participation in hygiene training positively associated with good practice (OR = 2.09, *p* = 0.015); 67.3% reported no need for further support, while others requested training, PPE, waste management, disinfectants, soap, and time resources.
Eigenstetter et al. (19)	Europe/Asia, Germany (Presentation in Singapore)	Cross-sectional study (presentation format)	Hospitals (19 hospitals), hospital cleaning staff ng = 1,447 (all cleaning workers), na = 214 (answered)	Systematic description of the working conditions of cleaning workers in German hospitals, with a focus on employee safety/self-protection and patient safety/hygiene. Keywords: None	Demographic variables, working equipment, work organization, knowledge, e.g. Infections, other workplace hazards, self-protection (e.g., use of protective gloves), hygiene (e.g., hand hygiene, indications for hand hygiene)	Results from nine cleaning staff showed 95.75% received protective gloves from their employer; glove materials used included chemical-resistant gloves (52.8%), nitrile or latex (37.3%), polypropylene (2.4%), and vinyl (1.9%); in 5.64% of cases, glove type was not reported; reasons for glove use included infection prevention (31.6%), skin protection (34.4%), warning by supervisor (9.9%), and personal initiative without warning (32.5%); hand disinfection was performed by 4 of 9 cleaning staff, with only 1 of 9 completing all six disinfection steps; timing of hand hygiene reported as after room cleaning (73.1%), before donning gloves (59.0%), at end of workday (68.9%), before cleaning (45.3%), after contact with blood or excreta (49.5%), never (1.4%), and in other situations (15.1%).
Elling et al. (20)	Africa, Republic of Malawi, Southeast Africa	Cross-sectional study, collection of qualitative and quantitative data	Healthcare facilities (44 facilities), cleaning staff (*n* = 57)	Investigation of the role of cleaning staff in preventing nosocomial infections and ensuring a clean environment in healthcare settings (in low-income countries). Keywords: cleaners, infection prevention and control, Malawi, water, sanitation and hygiene (WaSH), healthcare facilities.	Components of the SEIPS model (Systems Engineering Initiative for Patient Safety model), e.g., Work system [1. Person (e.g., education, training), 2. Organization, 3. Tools and technology (e.g., availability of supplies and resources) (including PPE), 4. Tasks, 5. Environment (e g., water availability, work-related injuries)].	Cleaners emphasized the importance of IPC measures, particularly hand hygiene, appropriate PPE use, safe medical waste disposal, and overall cleanliness; facilitating factors included cooperation with medical staff, job satisfaction, and equipment availability, while limiting factors included inadequate IPC training, additional tasks, occupational injury risk, workplace disrespect, and lack of incentives; 76% of ward staff (*n* = 29) and 53% of field staff (*n* = 10) attended 1 day introductory IPC training, with 39% (*n* = 15) and 37% (*n* = 7) receiving refresher training; cleaners reported absence of monitoring for HHC 89% (*n* = 51) reported lacking PPE such as gloves, goggles, aprons, and boots, citing financial limitations as the reason for shortages; despite this, 88% (*n* = 50) indicated that gloves and aprons were always available; hand hygiene resources were insufficient—soap lacking in 42% (*n* = 24) and hand-drying materials in 65% (*n* = 37); many cleaners resorted to air-drying or using personal towels due to limited access to water, soap, and drying materials, making effective hand hygiene sometimes impossible.
Kigozi et al. (21)	Africa, Uganda (Kampala)	Cross-sectional study (quantitative)	Hospital, cleaning staff (*n* = 120)	Recording the knowledge and practices of cleaning staff in relation to IPC, thereby creating a basis for decision-making for future improvement measures. Keywords: cleaners, cleaning, Infection prevention and control, knowledge, Training.	Three thematic sections: Section 1: Sociographic data, Section 2: Knowledge of IPC, Section 3: Practices in the field of IPC	Of 120 cleaners, 82 (68.3%) had completed IPC training and 70 (58.3%) demonstrated good IPC knowledge; average knowledge scores were higher among female cleaners (19.5/38.3) compared to males, and among operating room staff (20.5/59.4) compared to ward and outpatient staff; higher knowledge scores were also found in cleaners with university degrees (20.5/77.6), ≥2 years of experience (20.7/76.0), and those trained in IPC (19.6/76.0); professional experience positively correlated with IPC knowledge—cleaners with >5 years of experience were 10 times more likely to have good knowledge than those with < 1 year (aOR = 10.3, *p* = 0.006, 95% CI: 2.54); 46 cleaners (38.3%) washed hands with soap and water for 40–60 s as recommended, 51 (42.5%) for < 40 s, and 23 (19.2%) for >60 s; 111 (92.5%) wore heavy-duty gloves during cleaning; only 37 (30.8%) demonstrated good IPC practices (average score 14.3), with women (14.4/28.5) and operating room cleaners (15.2/43.9) achieving higher practice scores; multivariate analysis showed a significant positive correlation between knowledge and practice (*p* < 0.001).
Pirincci and Altun (22)	Europe/Asia, Turkey (Elazig)	Cross-sectional study (descriptive research design)	Hospital, cleaning staff (ng = 138)	Investigation of attitudes and behaviors regarding hand hygiene and cleaning. Keywords: cleaning staff, hand hygiene, cleaning, hospital	Sociodemographic data, attitudes toward infection prevention, education and training on work-related topics, hand hygiene, vaccinations, puncture and cut injuries.	In response to the question on the most effective infection prevention practices, 26.1% (*n* = 36) identified handwashing, 51.4% (*n* = 71) general hygiene, 10.1% (*n* = 14) careful equipment use, and 12.3% (*n* = 17) gave no response; gender comparison showed handwashing before work in 38.1% of men (*n* = 37) and 51.2% of women (*n* = 21) (*p* = 0.188), after work in 64.9% of men (*n* = 63) and 61.0% of women (*n* = 25) (*p* = 0.701), and after restroom use in 100% of both genders; hand hygiene by educational level showed significant differences—before work: 29.8% among elementary/low education (*n* = 28) vs. 68.2% among high school/higher education (*n* = 30) (*p* = 0.0001); after work: 54.3% (*n* = 51) vs. 34.1% (*n* = 37) (*p* = 0.0001); after restroom use: 100% in both groups; employment length analysis showed before work: 66.7% for ≤ 5 years (*n* = 12), 43.1% for 6–10 years (*n* = 28), and 32.7% for ≥11 years (*n* = 18) (*p* = 0.039); after work: 66.7% (*n* = 12), 69.2% (*n* = 45), and 56.4% (*n* = 31) (*p* = 0.331); after restroom use: 100% across all groups.
Sehbal Yesilbas et al. (23)	Europe, Asia, Turkey (Mugla)	Cross-sectional study, (questionnaire)	Hospital, hospital cleaning staff (*n* = 257)	Determination of current compliance with standard precautions of hospital hygiene and relevant influencing factors. Keywords: hospital, hygiene, standard precautions, healthcare associated infections	Sociodemographic data, working conditions, hygiene training, content of the “Standard Precautions Scale of Hospital Hygiene: Version of Cleaning Staff (HHSP)”	86.0% of cleaners reported receiving regular hygiene training, with 35.0% receiving it annually; 85.4% considered the training sufficient; overall mean HHSP score was 89.0 ± 6.6; HHSP scores were significantly higher among cleaners with higher education compared to those with low or no education (*p* = 0.0017); participants regularly attending hospital hygiene courses achieved significantly higher mean scores than untrained employees (*p* = 0.013); mean hand hygiene subscale scores were higher among women than men (*p* = 0.08); employees in lower-risk departments sometimes scored higher on the hand hygiene subscale than those in very high-, high-, or medium-risk areas (*p* = 0.018); correlation analysis revealed a very weak positive correlation between age and overall HHSP score (*r* = 0.163, *p* = 0.011), hand hygiene subscale (*r* = 0.163, *p* = 0.011), and general hygiene subscale (*r* = 0.161, *p* = 0.013).
Sendall et al. (24)	Australia (Queensland, Brisbane)	Cross-sectional study (Qualitative explorative design)	Hospital, hospital cleaning staff (ng = 12, focus group discussion round 1: 8 cleaners, focus group discussion round 2: 4 cleaners).	To determine the attitudes of cleaning staff toward hand hygiene and the national hand hygiene initiative. Keywords: hand hygiene; hospital cleaners; infection control; qualitative research; attitudes	Sociodemographic data, relevance and personal value of hand hygiene, feasibility of hand hygiene, culture of hand hygiene, reminder and promotion of hand hygiene	Three thematic areas were identified regarding hand hygiene among cleaning staff: (1) Hand hygiene culture: characterized by colleague support, managerial recognition, competition and incentives, and increasing importance of hand hygiene over the past 10–15 years; cleaning staff regularly participated in training and audits with feedback perceived as helpful; hand hygiene practiced for self-protection and respect for patients. (2) Reminders and promotion: confusion caused by numerous non-specific information events led to habituation effects; staff preferred simple, understandable reminders with concrete content, online modules, posters, and complementary activities accessible to all language levels. (3) Personal value of hand hygiene: workplace practices influenced personal hygiene behavior outside work; increased awareness and reflection on hand hygiene contributed to the development of good habitual practices and inspired staff to suggest innovative hygiene improvements.
Tolera et al. (25)	Africa, Ethiopia (Eastern Ethiopia)	Cross-sectional study	Hospitals (8 hospitals), sanitary workers (*n* = 809 –> 729 (90.11%) of them responded, IPC experts: *n* = 9)	Investigation of IPC compliance and relevant influencing factors. Keywords: compliance, control, determinants, hospital, infection prevention, sanitary workers	Sociodemographic data, compliance and determinants of IPC practice, compliance of IPC practice by hospitals, IPC environment	Overall IPC compliance was 36.21%; higher compliance observed under conditions of no heavy workload [AOR = 2.74, 95% CI (1.56–4.82)], low stress [AOR = 1.46, 95% CI (0.86–2.48)], working less than 8 h per day [AOR = 1.46, 95% CI (0.92–2.30)], and when work performance was socially recognized within the hospital [AOR = 6.08, 95% CI (4.24–8.71)]; shortage of qualified staff hindered safe IPC practices including training, monitoring, and feedback on hand hygiene; limited access to drinking water, hand hygiene facilities, and disinfected or sterilized containers prevented implementation of WHO-recommended IPC measures.
Yamazhan et al. (26)	Europe, Asia, Turkey (Izmir)	Cross-sectional study (questionnaire divided into two parts)	Hospital, Hospital cleaning staff (total 290 cleaners, ng = 240)	Evaluation of the knowledge of hospital cleaning Staff about Prevention of Nosocomial Infections. Keywords: hospital cleaning staff, nosocomial infection, knowledge level, questionnaire	Part 1 - Sociodemographic data, Part 2 - Questions assessing knowledge regarding nosocomial infections (e.g., cleaning procedures, hand hygiene, use of protective gloves, waste disposal)	Of 240 cleaning staff, 71.3% had received training on nosocomial infection prevention before starting work; no statistically significant difference in mean knowledge scores between trained (18.3 ± 4.2) and untrained (17.7 ± 3.2) staff (*p* = 0.294); correct responses on specific knowledge items included: spreading pathogens without illness (82.1%), recognizing hospital cleaners as a high-risk group (54.2%), prevention through handwashing after cleaning (58.8%), adherence to handwashing rules (80.8%), and glove use (90.4%); comparison with data from a 2004 observational study indicated discrepancies; findings underline the need for comprehensive induction and structured training programs by qualified personnel to improve consistent IPC and hand hygiene practices.
Yanke et al. (27)	United States of America (Wisconsin, Pennsylvania)	Cross-sectional study (qualitative-descriptive design, focus group discussion)	Hospital, environmental service workers (ESW), *n* = 7	Identification of work-system inhibiting and promoting factors with regard to the adherence to a CDI prevention measure package, from the perspective of the ESWs using the SEIPS approach (a novel approach based on the Human Factors Engineering Initiative). Keywords: infection prevention, environmental service workers, environmental management, bundle, qualitative research, clostridium difficile, human factors, focus group, veterans' affairs	Focus group: Hand hygiene, contact isolation precautions, environmental management. Collected data were thematically categorized using the SEIPS model: Person, organization, tools, tasks, and environment.	Environmental service workers (ESWs) demonstrated good knowledge of hand hygiene related to Clostridioides difficile infections (CDI) within the personnel component of the SEIPS model; awareness existed regarding the importance of handwashing and the limitations of alcohol-based hand disinfection against C. diff spores; ESWs expressed concern that handwashing duration was insufficient to ensure safe spore removal; availability of hand hygiene materials such as sinks and soap was identified as essential to promote compliance; concerns were raised about the lack of awareness among other professional groups regarding alcohol-based hand disinfection limitations; organizational and hierarchical barriers caused discomfort when addressing hand hygiene non-compliance by physicians.
Toffolutti et al. (28)	Europe, United Kingdom (various regions)	Cross-sectional study	Hospitals (126 hospitals), hospital cleaning staff	Investigating the relationship between hospital cleaning outsourcing, MRSA incidence, perceived cleanliness, availability of hand hygiene equipment, and cost reduction. Keywords: outsourcing, hospital acquired infections, hospital cleaning, contracting-out	Hospital incidence of MRSA per 100,000 hospital bed-days (Public Health England Annual Reports, 2015), Patient reports on cleanliness, cleanliness of patient rooms, staff reports on the availability of handwashing, the availability of hand hygiene facilities and equipment. Data on outsourcing/non-outsourcing of cleaning	Comparison of hospitals with external vs. internal cleaning showed an average MRSA incidence of 2.28 per 100,000 bed-days for externally cleaned hospitals and 1.46 per 100,000 bed-days for internally cleaned hospitals; sufficient handwashing supplies were reported by 63.0% of staff in hospitals with external cleaning and 68.0% in hospitals with internal cleaning (*t*-test = 3.47, *p* ≤ 0.001); outsourced cleaning was associated with approximately 1.22% fewer staff having easy access to handwashing supplies (95% CI: −1.79% to 0.58%); availability of handwashing detergent was reported by 61.3% of employees in hospitals with external cleaning and 62.7% in hospitals with internal cleaning.
Chen et al. (29)	Asia, China (13 provinces)	Methodological study	Hospitals, environmental service workers (ESW) (*n* = 1,176)	Development and validation of a questionnaire to assess the attitudes, knowledge, behaviors, and experiences of environmental service workers (ESWs) with regard to IPC in hospitals. Keywords: infection prevention and control, environmental service worker, knowledge, attitude, practice, experience, Delphi method	1. Sociodemographic data, 2. KAPE-IPC-Q items - Topic-related items: Knowledge (e.g., hand hygiene, correct use of cleaning equipment, correct use of PPE), Attitudes (e.g., regarding the relevance of surface and hand hygiene), Practice (e.g., wearing PPE, hand hygiene measures, infection symptoms), Experience (e.g., regular and target group-specific training, equipment and PPE, work controls).	Development of the KAPE-IPC-Q questionnaire addressing knowledge, attitudes, practices, and experiences on IPC topics including surface cleaning, disinfection, isolation, hand hygiene, occupational health, and COVID-19; first Delphi round with 16 experts (15 responses) resulted in exclusion of 16 items, modification of 31, merging of two, and addition of nine; remaining 33 items showed CVV ≤ 0.25 (Kendall's W correlation = 0.204/0.128, importance W = 0.249/0.142, *p* < 0.001); second Delphi round with 15 experts confirmed CVV ≤ 0.25, excluded one item, modified nine (Kendall's W correlation = 0.221/0.096, importance W = 0.22/0.101, *p* < 0.05); final version comprised four first-level indices (KAP, experience) and 49 second-level indices; pilot survey with 1,176 participants demonstrated high reliability (Cronbach's α = 0.967, S-CVI/mean = 0.989).
Demirel and Ozdemir (30)	Europe/Asia, Turkey (Ankara)	Methodological study	Hospital, hospital cleaning staff (*n* = 330)	Development of a scale for determining hospital hygiene standards for the cleaning staff profession. Keywords: cleaning staff, infection control, occupational health, standard precautions.	Sociodemografic data, the five thematic sub-dimensions of the scale: 1. General Cleaning, 2. Waste Management, 3. Personal precaution, 4. Hand hygiene (e.g., handwashing situations during daily work, use of protective gloves, Items relating to the hand hygiene behavior of cleaning staff) 5. PPE use.	The Hand Hygiene Practice Scale (HHPS) comprises five dimensions and 30 items, with factor loadings between 0.86 and 0.32 and a total explained variance of 51.16%; confirmatory factor analysis supported the five-dimensional structure; hand hygiene–related items include washing visibly and non-visibly soiled hands, handwashing before and after donning and doffing protective gloves, washing hands before cleaning activities, using only clean and hygienic gloves, checking and covering hand wounds before glove use, and wearing gloves when preparing isolation rooms.
Tesfaye et al. (31)	Africa, Ethiopia (Gondar City)	Cross-sectional study (Institutional)	Healthcare facilities, healthcare cleaning staff (*ng* = 390)	Investigation of IPC and associated factors. Keywords: healthcare-associated infection, infection prevention, healthcare cleaners, Ethiopia	Sociodemographic data, behavioral and organizational factors (e.g., IPC training, presence and participation in IPC committees, availability of hand hygiene materials, vaccinations, PPE), knowledge about IPC (e.g., IPC principles, disinfection, nosocomial infections, hand hygiene), IPC (moments of hand hygiene in the work context, IPC guidelines, disposal, vaccinations)	Of 390 surveyed cleaners, 205 (52.6%) received IPC training within the last 12 months; 31.5% (*n* = 123) reported the existence of an IPC committee, and 11.5% (*n* = 45) participated in or collaborated with one; workplace compliance with IPC guidelines was confirmed by 49.0% (*n* = 191); soap and alcohol-based hand sanitizer were used by 75.4% (*n* = 294); 47.7% (*n* = 186) demonstrated good IPC knowledge, while 52.3% (*n* = 204) had poor knowledge; 78.2% (*n* = 305), 76.7% (*n* = 299), and 78.7% (*n* = 307) knew that disinfection, PPE use, and hand hygiene prevent nosocomial infections; 63.1% (*n* = 246) had good knowledge of hand hygiene, and 80% (*n* = 312) knew in which situations hand hygiene is required; 68.7% (*n* = 268) recognized the equivalence of hand disinfection and handwashing with visibly clean hands; overall, 52.3% demonstrated good IPC practices; 73.3% (*n* = 286) performed hand hygiene after contact with medical waste; 43.3% (*n* = 169) practiced proper hand drying to prevent recontamination, and 40.0% (*n* = 156) maintained short, clean nails without nail polish; logistic regression analysis showed that cleaners with good IPC knowledge were 1.56 times more likely to exhibit good IPC practices [AOR = 1.56, 95% CI (1.03–2.37)]; availability of IPC guidelines in the workplace increased the likelihood of good practices by 1.54 times (AOR = 1.54, 95% CI (1.01–2.33)].

CDI, Clostridioides difficile infection; ESW, Environmental Service Worker; HHC, Hand hygiene compliance; HAI, Healthcare- associated infections; HHPS, Hand Hygiene Practice Scale; HHSP, Hospital Hygiene Standard Precautions; PPE, Personal Protective Equipment; SEIPS model, Systems Engineering Initiative for Patient Safety; Spp., Species pluaralis; WaSH, Water Sanitation Hygiene; WHO, Word Health Organization.

#### 3.3.1 KAP of cleaning staff regarding hand hygiene

A total of 16 studies addressing the topics of KAP regarding IPC in general, and specifically the hand hygiene of cleaning staff in healthcare facilities, were identified. The content of the studies focusing on the current situation regarding IPC in general and hand hygiene among cleaning staff in particular is taken into account (see Table 2). Using 12 self-formulated items (Items A to L), the findings from the included studies were systematically recorded and presented in a matrix.

Table 3 shows that 13 of the 16 studies address hand hygiene in general (17, 18, 20–27, 30, 31). One study dealt exclusively with handwashing (30), while eight others addressed both hand hygiene in general and handwashing (20–22, 24–26, 30, 31). One study focused exclusively on hygienic alcohol-based hand disinfection (19). Another study addressed both handwashing and hygienic hand disinfection (16). One study addressed general hand hygiene, handwashing, and hygienic hand disinfection (27). Fourteen of the 16 included studies addressed KAP related to IPC in general and specifically regarding the hand hygiene of cleaning staff (16, 18–27, 29–31). Three studies addressed participation in the development and delivery of training measures (18, 24, 31). Twelve studies addressed the determination of the influence of training measures on IPC and hand hygiene behavior (16, 18, 20, 21, 23–27, 29–31).

**Table 3:** Overview matrix of relevant content from the included studies on KAP regarding hand hygiene (as a single aspect or aspect of IPC) among cleaning staff.

Item	Assigned study numbers →	16	17	18	19	20	21	22	23	24	25	26	27	18	29	30	31
A	Focus on hand hygiene or IPC (in general)?	IPC	HaHy	IPC	IPC	IPC	IPC	IPC	IPC	HaHy	IPC	IPC	IPC^*^	IPC	IPC	IPC	IPC
B	Focus on hygienic hand disinfection, handwashing, or hand hygiene in general?	HW, HD	HaHy	HaHy	HD	HaHy, HW	HaHy, HW	HaHy, HW	HaHy	HaHy, HW	HaHy, HW	HaHy, HW	HaHy, HW, HD	HaHy, HW	HaHy	HaHy, HW	HaHy, HW
C	Focus on recording the KAP of the cleaning staff?	✓	X	✓	✓	✓	✓	✓	✓	✓	✓	✓	✓	X	✓	X	✓
D	Mention and/or record of cleaning staff attending and participating in IPC training and hand hygiene training?	X	X	✓	X	X	X	X	X	✓	X	X	X	X	X	X	✓^*^
E	Investigation of the effects of training measures on IPC and hand hygiene behavior?	✓	X	✓	X	✓	✓	X	✓	✓	✓	✓	✓	X	✓^**^	X	✓
F	Assessing the availability and accessibility of IPC equipment and hand hygiene equipment?	X	X	✓	X	✓	X	X	X	X	✓	X	✓	✓	X	✓	X
G	Assessment of the hygienic condition of the hands (through microbiological testing)?	X	✓	X	X	X	X	X	X	X	X	X	X	X	X	X	X
H	Recording information about nail hygiene, jewelry and wound hygiene during working hours on fingers and forearms?	X	X	✓	X	X	X	X	X	X	X	X	X	X	X	X	✓
I	Recording the use of protective gloves?	✓	X	X	✓	✓	✓	X	X	X	X	✓	X	X	✓	X	✓
J	Recording hand hygiene practices, taking into account the moments when hand hygiene should be performed?	✓	X	X	✓	X	X	✓	X	X	X	✓	✓	X	✓	X	✓
	Before putting on the protective gloves	X			✓			X				X	X		X		X
	Before cleaning work	X			X			✓				X	X		X		X
	After contact with potentially infectious surfaces	X			X			X				X	✓		X		X
	After contact with potentially infectious material	X			✓			X				X	X		X		X
	After contact with medical or potentially infectious waste	✓			X			X				X	X		X		✓
	In case of visible contamination of the hands	X			X			X				X	X		X		✓
	In cases of non-visible contamination of the hands	X			X			X				X	✓		X		X
	After using the bathroom	X			X			✓				X	X		X		X
	After removing the protective gloves	X			X			X				X	X		X		X
	After cleaning work	X			✓			✓				✓	X		X		X
	Other situations	X			✓			X				X	X		X		X
K	Recording individual steps of hand hygiene procedures?	X	X	X	✓	X	X	X	X	X	X	X	X	X	X	X	X
L	Recording of hand hygiene measures in relation to specific pathogens?	X	X	X	X	X	X	X	X	X	X	X	✓^***^		X	X	X

^*^Cooperation and exchange between cleaning staff and employees of the IPC department.

^**^Development and validation of a questionnaire to assess attitudes, knowledge, and behavior in relation to IPC and hand hygiene in particular, thereby also determining a correlation between training and behavior.

^***^In the context of a specific pathogen (Clostridioides difficile).

✓ Green background = Thematized; X / Red background = Not thematized; Gray, Irrelevant sub-question/follow-up question; HaHy, Hand hygiene; IPC, Infection prevention and control; KAP, Knowledge, attitude, practice; HD, Hand disinfection; HW, Handwashing.

The studies (29) and (30) are methodological studies describing the development and implementation of a questionnaire in order to assess KAP. Five of the 16 included studies assessed the availability and accessibility of IPC and hand hygiene equipment (18, 20, 25, 27, 30). One study used microbiological methods to assess hand hygiene (17). Three studies addressed the presence of factors potentially hindering hand hygiene (18, 30, 31). The studies addressed fingernail length, fingernail shape, the wearing of jewelry on fingers and forearms, and wound hygiene (18, 30, 31).

Eight of the 16 studies recorded the availability or use of protective gloves during working hours (16, 19–21, 26, 29–31).

Furthermore, hand hygiene practices taking into account work-related tasks were recorded (16, 19, 22, 26, 27, 29–31). The following 11 hand hygiene moments were considered: before putting on protective gloves (two studies (19, 30)), before cleaning (two studies (22, 30)), after contact with potentially contaminated surfaces (one study (27)), after contact with potentially infectious material (one study (19)), after contact with medical and potentially infectious waste (two studies (16, 31)), in the case of visible hand contamination (two studies (30, 31)), in the case of non-visible hand contamination (two studies (27, 30)), after using the bathroom (one study (22)), after removing protective gloves (one study (30)), after cleaning (three studies (19, 22, 26)), and in other situations (one study (19)). One study assessed adherence to the correct respective steps of a hand hygiene measure leading to even coating of the hands by the respective hand hygiene product; six individual steps were recorded (19). The selection and implementation of pathogen-specific hand hygiene measures were focus of a study in connection with the anaerobic, spore-forming bacterium *Clostridioides difficile* (27).

#### 3.3.2 Factors influencing hand hygiene

**Figure 2:**
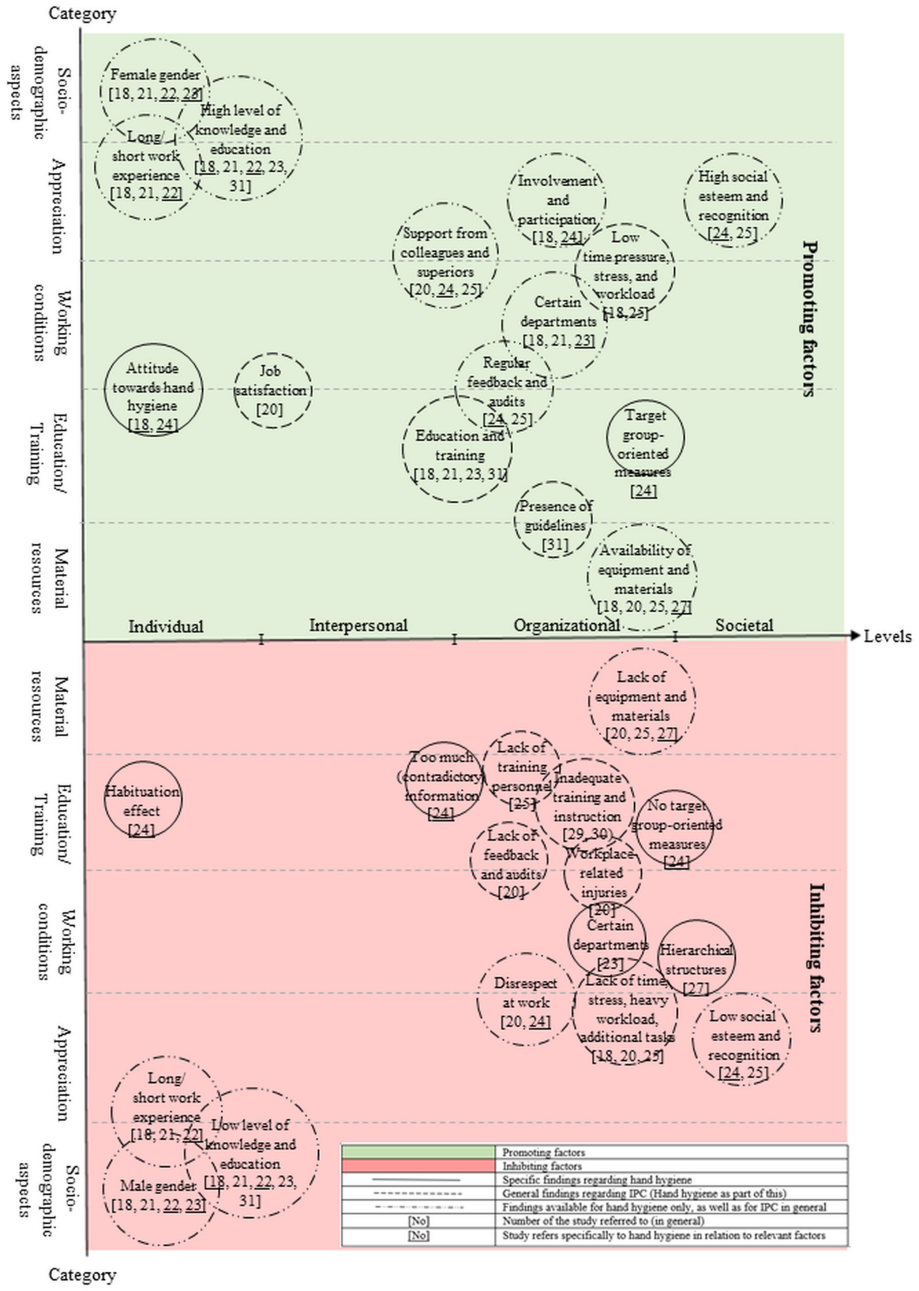
Bubble chart – Overview and classification of promoting and inhibiting factors.

Ten studies identified factors that either promote or inhibit the implementation of infection prevention principles with regard to IPC in general and hand hygiene among cleaning staff in particular (18, 20–25, 27, 30, 31). Figure 2 serves to graphically represent and classify the promoting and inhibiting factors.

The identified factors can be horizontally divided into four levels: (1) individual, (2) interpersonal, (3) organizational, and (4) societal. At the same time, these factors are vertically structured into five categories: (1) material resources, (2) education/training, (3) working conditions, (4) recognition, and (5) sociodemographic factors.

To improve clarity, Figure 2 distinguishes between findings exclusively relating to hand hygiene and those relating to IPC more generally. The different lines around the factors indicate whether the respective factor refers explicitly to knowledge of hand hygiene (solid line), general knowledge of IPC (dashed line), or both, i.e., explicit knowledge of hand hygiene and general knowledge of IPC (dashed and dotted lines). The underlined numbers in the figure indicate studies in which the respective influencing factor was explicitly investigated in connection to hand hygiene or its influence on hand hygiene, while the not underlined numbers indicate studies describing relevant influencing factors on IPC without specifically addressing hand hygiene. The following Figure 2, presents an overview and classification of the promoting and inhibiting factors identified in the included studies. The contents of Figure 2 are explained below.

A total of 15 promoting factors were identified, which can be classified at the individual, interpersonal, organizational and societal levels or between respective levels. The individual level includes the factors “positive attitude toward hand hygiene (18, 24),” “little or extensive professional experience (18, 21, 22),” and “female gender (18, 21–23).” The factors “job satisfaction (20)” and “high level of knowledge and education (18, 21–23, 31)” can be classified between the individual and interpersonal levels. There are no promoting factors assigned to the interpersonal level. The factors “support from colleagues and superiors (20, 24, 25)” and “training and further education (18, 21, 23, 31)” are classified between the interpersonal and organizational levels. The promoting factors at the organizational level include “availability of recommendations (31),” “regular feedback and audits (24, 25),” “work in specific areas and departments, such as work in surgery, pediatrics, obstetrics, and, to some extent, work in lower risk departments (18, 21, 23),” as well as “low time pressure, stress and workload (18, 25)” and “participation and involvement in training and the development of measures, particularly in the area of hand hygiene (18, 24).” The factors “access to materials and products, especially with regard to hand hygiene (18, 20, 25, 27)” and “target group-specific measures, especially with regard to hand hygiene (24)” are classified between the organizational and societal levels. One of the promoting factors at the societal level is “high social recognition and appreciation (24, 25).”

In addition, a total of 16 inhibiting factors were identified and classified at the individual, interpersonal, organizational, and societal levels or between levels. The individual level includes the factors “habituation effect (24),” “little or extensive work experience (18, 21, 22)” and “male gender (18, 21–23).” The factor “low level of knowledge and education (18, 21–23, 31)” can be classified between the individual and interpersonal levels. There are no inhibiting factors assigned to the interpersonal level. The factor “too much conflicting information about IPC in general and hand hygiene in particular (24)” can be classified between the interpersonal and organizational levels. The inhibiting factors “insufficient training and instruction (29, 30),” “lack of qualified and suitable training personnel (25),” “lack of feedback and audits (20),” “accidents at work (20),” “disrespect in the workplace (20, 24),” “work in certain areas and wards, e.g., some of the work in middle and high-risk departments (23)” and “lack of time, stress, high workload and additional tasks (18, 20, 25).” Between the organizational and societal levels, the factors “lack of equipment and materials, including for hand hygiene (20, 25, 27),” “no target group-specific measures (24)” and “hierarchical structures (27)” are classified. One of the inhibiting factors at the societal level is “low social esteem and recognition (24, 25).”

The graphical representation of the promoting and inhibiting factors shows a broadly comparable distribution. A large proportion of the identified factors can be located in the horizontal levels at the individual level (level 1) and the organizational level (level 3) and in the vertical categories of education/training (category 2), working conditions (category 3) and socio-demographic factors (category 5). Few factors are assigned to the horizontal levels interpersonal (level 2), societal (level 4) and the vertical category material resources (category 1). This can be deduced from the gaps in the graph.

### 3.4 Target group-specific interventions

A total of nine studies meeting the inclusion criteria and addressed hand hygiene interventions for cleaning staff were identified. Four of the included studies had a pre-post study design, two had a quasi-experimental study design, one had a combination of a prospective semi-experimental study design and a pre-post study design, one had a cross-sectional study design, and one was a case study review. The studies originated in Egypt, England, Saudi Arabia, Turkey, China, the USA (New York), Australia (Brisbane), Cambodia, Gambia, Myanmar, Tanzania, Nigeria, and Vietnam. Data collection methods included questionnaires (before, during, and after the intervention), interviews (structured, semi-structured), observations (direct, indirect, covert, and video-based), effectiveness evaluation tools, and literature reviews. The outcomes collected in the included studies can be divided into the following five categories: (1) sociodemographic data of the target group, (2) organizational data, e.g., Occupational health and safety, training measures, especially IPC training, ergonomics, (3) KAP on IPC- topics in general, especially with regard to hand hygiene (six new moments of hand hygiene, “one before, five after”), (4) microbiological, biochemical (ATP measurement) and epidemiological data. Two of the nine studies focus on hand hygiene interventions for cleaning staff (5, 6), while the remaining seven studies address IPC interventions for cleaning staff in healthcare facilities in general. Topics include general education and training, cleaning organization, occupational health and safety, personal hygiene, cleaning equipment, the use of PPE, surface cleaning and disinfection, and waste and laundry management. Table 4 lists information on author (year of publication), continent, country, study design, data collection, setting, population, sample size (n), thematic focus, relevant keywords, outcomes, and relevant key findings.

**Table 4:** Overview of studies on hand hygiene interventions hygiene (individual intervention or IPC intervention bundle) for cleaning staff.

References	Continent, country	Study design, data collection, observation period (after implementation of the intervention):	Setting, population, sample size (*n*)	Thematic focus, relevant keywords	Outcomes	Key findings
Chen et al. (6)	Asia, China (Wuhan)	Before-and-After-Study, Analysis and intervention study 1 month	Hospital, hospital cleaning staff (Observation: *n* = 50, Intervention: *n* = 20)	Examination of hand hygiene indicators and identification of opportunities for hand hygiene monitoring. Keywords (by the authors): healthcare-associated infection, hand hygiene, hand hygiene monitoring, cleaner, risk identification.	Hand hygiene indications according to the “once before, five times after” principle: (1) Before cleaning and disinfection, (2) After preparing cleaning equipment, (3). After cleaning and disinfection work, (4) After removing PPE, (5) After disposing of medical waste, 6. After contact with the patient environment.	Six key indications for hand hygiene among cleaning staff were identified: (1) before cleaning/disinfection, (2) after preparing tools, (3) after cleaning/disinfection, (4) after removing personal protective equipment, (5) after transporting medical waste, and (6) after contact with the patient environment; summarized as the “One Before, Five After” framework; 20 cleaning staff were selected and trained according to this principle; preliminary test phase results showed 89.60% (112/125) compliance and 91.20% (114/125) correctness of hand hygiene among trained staff.
He et al. (5)	Asia, China (Wuhan)	Quasi-experimental study 3 months	Hospital, hospital cleaning staff (ng1 = 141 –> selected: ng2 = 89), Four groups, each at four different locations, with no overlap in personnel [control group (ncontrol = 21), case group 1 (ncase1 = 26), case group 2 (ncase2 = 26), case group 3 (ncase3 = 18)]	Investigation of the effect of six new hand hygiene moments on HHC among hospital cleaning staff. Keywords: compliance, hand hygiene, healthcare, infection; intervention study	HHC (four groups) before the intervention, 1 month, 2 months and 3 months, after the intervention, compliance at different times and time distributions. Overall compliance was defined as the ratio of the number of hand hygiene measures actually performed (x) to the number of occasions (N) when a hand hygiene measure would have been necessary	Six target-group-specific hand hygiene moments: before cleaning and disinfection activities, after preparing cleaning equipment, after cleaning and disinfection activities, after removing protective clothing, after transporting or handling medical waste, after sorting waste. Control group: 106 hand hygiene measures before and 591 after a 3-month intervention; compliance decreased from 30.19% [95% CI (0.2227–0.395)] to 27.41% [95% CI (0.2397–0.3114)]; Case group 1: 115 measures before and 706 after; compliance increased from 31.30% [95% CI (0.2355–0.4026)] to 42.78% [95% CI (0.3918–0.4646)]; Case group 2: 524 measures before and 645 after; compliance rose from 23.85% [95% CI (0.2040–0.2768)] to 59.22% [95% CI (0.5538–0.6295)]; Case group 3: 591 measures before and 519 after; compliance improved from 27.41% [95% CI (0.2397–0.3114)] to 83.62% (95% CI (0.8019–0.8655)]; greatest improvement observed in case group 3 with 56.21% increase (*p* < 0.05); highest compliance in case group 2 occurred “after removing protective equipment” [64.77%, 95% CI (0.5436–0.7394)] and lowest “before cleaning and disinfection” [54.55%, 95% CI (0.4525–0.6354)]; in case group 3, highest compliance was “after transporting medical waste” [90.72%, 95% CI (0.833–0.9504)] and lowest “after preparing equipment” [78.33%, 95% CI (0.6638–0.8687)]; peak observation times for hand hygiene measures were between 6:00–9:00 a.m. and 2:00–3:00 p.m., with 154–182 observed actions.
Aziz (32)	Europe, England (Manchester)	Before-and-after study 2 months	Hospital, domestic staff in hospital (*n* = 50)	Study of the effect of a workbook for cleaning staff in hospitals on awareness of infection prevention and improvement of cleanliness Keywords: infection prevention and control, education and training, domestic staff, workbook.	Sociodemographic data, questions about training, using the workbook, infection prevention (e.g., hand hygiene, needle stick and cut injuries, use of PPE) clinical audit scores pre/post infection control workbook for domestics.	Before the intervention, 58% (*n* = 22) of employees had received IPC training at job start, and the hand hygiene score was 68%; 34% (*n* = 13) expressed a desire for IPC training; after the intervention, hand hygiene improved to 95%, 95% of participants requested IPC training, and 76% expressed interest in training updates; contact with the IPC team increased from 5% to 86%; clinical audits covering 10 areas showed pre-intervention scores between 58% and 81%, increasing to over 95% post-intervention (except two wards scoring 91% and 93%).
Battan et al. (33)	Asia, Saudi Arabia	Before-and-after study 6 months	Hospital, hospital housekeeping staff (*n* = 124)	Investigation of the effectiveness of simulation-based training for housekeeping staff in hospitals. Keywords: simulation technology, personal protective equipment, hand hygiene, biological spill kit, terminal cleaning, housekeeping staff.	Sociodemographic data, work-related topics: (1) General knowledge assessment, (2) Hand hygiene, (3) Biological spills, (4) PPE, (5) Terminal cleaning	Results showed a 33% improvement in general knowledge and a 53% improvement in hand hygiene following the training program; paired two-sample *t*-test results indicated significant increases in mean scores: general knowledge – observations: 116, before: 7.30, after: 9.72 (*p* ≤ 0.000); hand hygiene – observations: 116, before: 6.30, after: 9.76 (p ≤ 0.000); patient satisfaction with hospital cleaning improved from 86.8% pre-intervention to 97.13% post-intervention.
Attia et al. (34)	Asia, Africa, Egypt (Zagazig)	Quasi-experimental study, three instruments were used 7 months (March 2021–September 2021)	Hospital, hospital housekeepers (*n* = 68, ncase = 34, ncontrol = 34)	Assessment of the effectiveness and impact of health education measures relating to occupational health risks among housekeeping staff in hospitals. Keywords: body mechanics techniques, housekeeping workers, occupational health hazards, universal precautions, workplace hazards, Zagazig University hospitals	(1) Questionnaire on socio-demographic data, hazards and health, occupational health and safety measures (2) Body mechanics skills checklist (3) Handwashing checklist (Assessment of routine handwashing technique, observation checklist, without knowledge of the person being observed, used as a pre-test, post-test, and follow-up format)	In the study population, correct handwashing performance increased from 8.8% (3 of 34) before intervention to 97.1% (33 of 34) after and 94.1% (32 of 34) at follow-up (*p* < 0.001); in the control group, performance remained unchanged at 2.9% (1 of 34) across all phases; a significant positive correlation was found between overall scores for knowledge, body mechanics, and handwashing performance across study phases; correlations between body mechanics and handwashing performance were statistically significant in all phases (*p* < 0.001); a further significant positive correlation was observed between staff knowledge and handwashing performance (*p* = 0.006).
Bulut et al. (35)	Europe, Asia, Turkey	Semiexperimental type, before-and-after-study 3 months	Hospital, hospital cleaning staff (*n* = 94)	Determining the effectiveness of a training program on infection prevention and personal hygiene for cleaning staff in hospitals. Keywords: cleaning staff, hygiene, before and after training.	Sociodemographic data, hygiene behavior e.g., personal hygiene (body, hair and face wash, dental hygiene, eye cleaning, hand hygiene, e.g., handwashing, cutting fingernails, use of soap and towels)	The training program had an overall positive effect on knowledge and behavior regarding personal hygiene; changes in hand hygiene practices included water only: before 1 (1.1%), after 1 (1.1%); alcoholic solution: before 11 (11.7%), after 11 (11.7%); water only for 15–20 s: before 30 (31.9%), after 24 (25.5%); soap and water: before 52 (55.3%), after 58 (61.7%) (*p* = 0.401); changes in fingernail and toenail cutting patterns were not statistically significant—both straight: before 44 (46.8%), after 44 (46.8%); both round: before 17 (18.1%), after 23 (24.5%); fingernails rounded/toenails straight: before 24 (25.5%), after 19 (20.2%); fingernails straight/toenails rounded: before 9 (9.6%), after 8 (8.5%) (*p* = 0.270); preference for soap type shifted slightly—bar soap: before 37 (39.4%), after 30 (31.9%); liquid soap: before 57 (60.6%), after 64 (68.1%) (*p* = 0.143).
Martin et al. (36)	United States of America (New York)	Prospective, quasi-experimental before-and-after intervention study 3 months and 1 year later	Hospitals (5 hospitals), environmental service workers (ESW) (*n* average = 357, between 303 and 391 participants/program component)	Development and implementation of a five-part training program for environmental service workers on the topic of infection prevention. Keywords: None	HAI, Hand hygiene, Isolation measures and PPE, Daily cleaning measures, The assessment of the effectiveness of training program: questionnaires (ARS questions) cleaning checks (including 3M Clean Trace –>Surfaces were considered clean at a value of < 250 RLU)	The training program comprised five modules with an average of 357 participants per module (range 303–391) and a total of 1,740 participants; 95% reported that the concrete solutions for overcoming work-related barriers were helpful; post-intervention results showed improved understanding of infection transmission routes (98%), increased confidence in donning and doffing protective clothing (96%), improved understanding of disinfecting frequently touched surfaces (96%), and greater confidence in performing hand hygiene measures (96%); comparable learning outcomes were observed across all hospitals; a significant 11% reduction in the CDI SIR for hospital admissions was recorded (*p* = 0.03), while a 23% reduction in the MRSA-BSI SIR was not statistically significant (*p* = 0.22).
Mitchell et al. (37)	Australia (Brisbane)	Cross-sectional study integrated into a stepped wedge study Establishment: 1 month, Control: 2 months, Intervention: 5 months−1 year	Hospitals, Environmental Service Workers (ESW) (*n* = 807).	REACH study: Efficacy assessment of a multimodal cleaning intervention. Keywords: cross infection, infection control, hospitals, health services, housekeeping, maintenance, translational research, environment	Sociodemographic data, knowledge and reported practice (IPC –> nosocomial infections, hand hygiene, isolation measures, identification of common skin and hand contact sites), attitudes, role as a cleaner, perceived organizational support	Before the intervention, 494 (80.2%) of cleaning staff had already received training; a positive trend was observed in correct responses regarding hand hygiene (*p* = 0.07), use of protective gloves (*p* = 0.09), and PPE use for isolated patients (*p* = 0.09); several key topics, including infection transmission routes, glove use, waste management, and working in isolation rooms, showed high baseline knowledge levels (>95% pre-test); hand hygiene question results showed prevalence of hand contamination awareness increasing from 95.79% (*n* = 595) to 97.94% (*n* = 291) (*p* = 0.07); correct identification of glove use situations remained high—when removing waste: 99.35% (*n* = 612) to 99.66% (*n* = 296) (*p* = 0.50); when working in isolation rooms: 99.67% (*n* = 306) to 99.32% (*n* = 293) (*p* = 0.51); during lunch: 95.51% (*n* = 557) to 92.36% (*n* = 275) (*p* = 0.09).
Patrick et al. (38)	Asia, Africa, Initiative 1 (WHO): Cambodia, Gambia, Myanmar, Tanzania Initiative 2 (CDC): Nigeria (Lagos), Vietnam (Ho Chi Minh City)	Commentary, Collection of experience reports Not specified	Healthcare facilities, cleaning staff.	Insights into facilitating and inhibiting factors and practical experiences in the implementation of hospital cleaning and infection prevention initiatives. Keywords: healthcare environment, cleaning, cleaners, infection prevention, training, resources, Leadership, Investment, Adaptation, Professionalization	Initiative 1 (WHO): Trainers' guide (prepare, deliver, sustain), modules and resources (teach, visualize, check). Linking components e.g., “organization,” “policies and procedures,” “staffing and training,” “infrastructure and supplies,” and “monitoring, feedback, and audit.” Initiative 2 (CDC): 5-step implementation cycle: 1. A - Prepare for action 2. B - Conduct baseline assessment 3. C - Develop action plan 4. D - Implement program improvements 5. E - Evaluate impact/sustain.	Three main barriers to effective IPC and hand hygiene practices among cleaning staff were identified: (1) Low competence and professional status: predominantly female workforce with low education and literacy levels, limited decision-making power; proposed solutions included visual and video-based, language-adapted, and context-specific training materials tailored to the target group. (2) Low pay and high workload: insufficient working hours, high staff turnover, and perception of training as an additional burden; suggested solutions included delegating cleaning tasks to nurses or support staff, prioritizing cleaning plans, and offering modular ward-based training to reduce costs and turnover. (3) Limited interaction with IPC teams: lack of integration of cleaning staff into IPC and patient safety structures and unclear responsibilities; recommended measures included management commitment to IPC team collaboration, use of “champions” or multipliers, continuous support through tools such as checklists and fluorescent gel, and joint revision of cleaning protocols.

ARS questions, Assessment of the effectiveness of training program questionnaires; CDC, Centers for Disease Control and Prevention; CDI SIR, Clostridioides difficile Infections Standardized Infection Ratios; ESW, Environmental Service Workers; HAI, Healthcare/ Hospital Acquired Infection; HHC, Hand hygiene compliance; IPC, Infection Prevention and Control; MRSA BSI SIR, Methicillin resistant Staphylococcus aureus Blood Stream Infections Standardized Infection Ratios; ncase, ncase; ncontrol, Random sample control; PPE, Personal Protective Equipment; REACH study, Researching Effective Approaches to Cleaning in Hospitals; RLU, Relative light units; WHO, World Health Organization.

**Figure 3:**
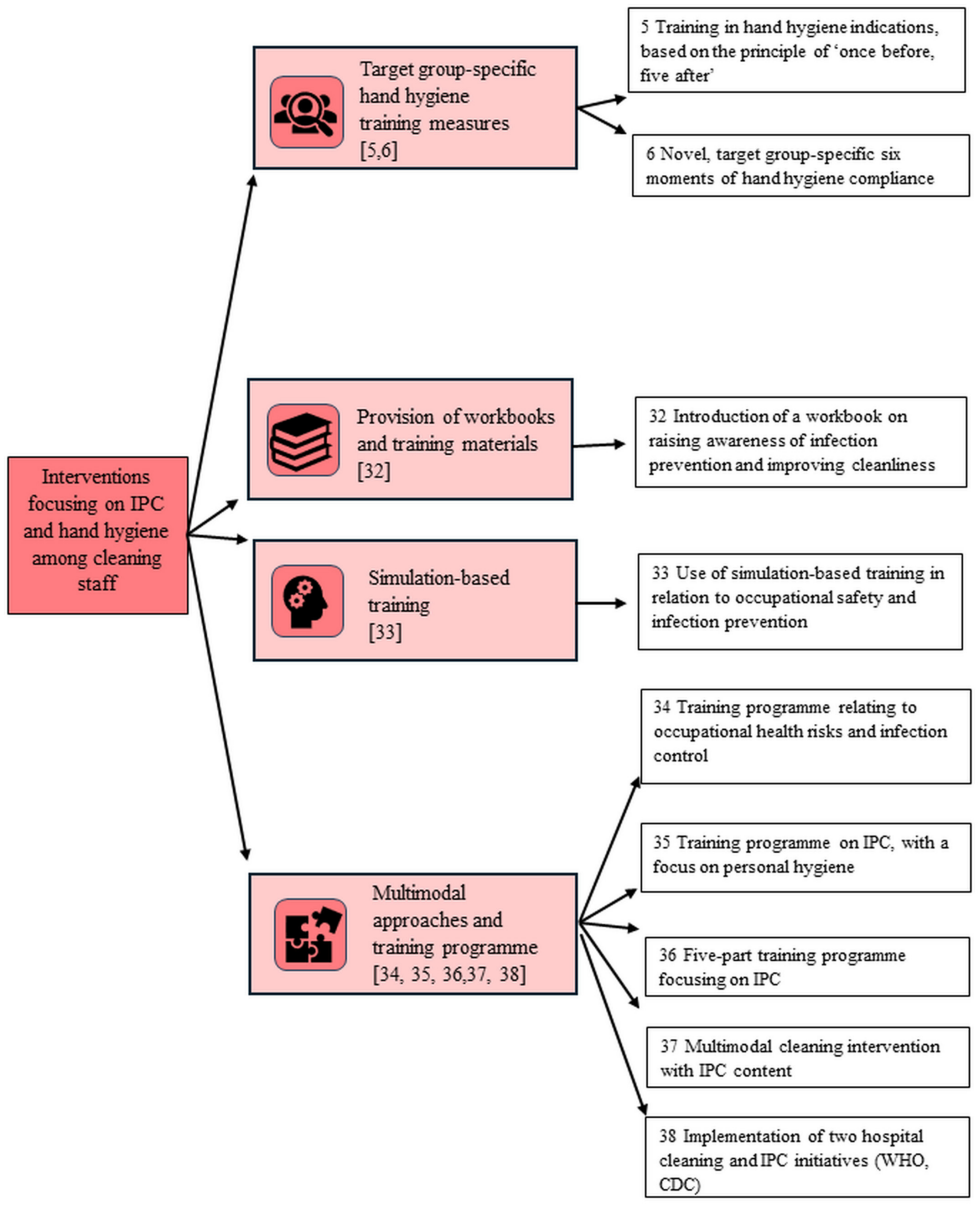
Concept map, overview and classification of identified interventions for hand hygiene (individual intervention or IPC intervention bundle) among cleaning staff.

The subsequent Figure 3, serves to visualize the subdivision and classification of the identified interventions, based on a concept map. The content of Figure 3 is explained in the following section.

The identified interventions can be divided into the following four categories: (1) Target group-specific hand hygiene training measures, (2) Provision of workbooks and training material, (3) Simulation-based training, (4) Multimodal approaches and training programs. Category 1, “Target group-specific hand hygiene training measures,” comprises two studies (5, 6). Taking into account target group-specific activities, hand hygiene moments are redefined, theoretical and practical training is provided, and the effectiveness of the interventions is determined through before-and-after comparisons (6) or comparisons with control groups (5). Category 2, “Provision of workbooks and training material,” comprises one study (32). As part of the intervention, a workbook focusing on infection prevention and environmental hygiene, based on individual topics such as hand hygiene, puncture and cut injuries, and the use of PPE, is implemented, and its effect is determined through a before-and-after comparison. Category 3, “Simulation-based training,” includes one study (33). This simulation-based training for cleaning staff, focusing on infection prevention (including hand hygiene, biological spills, PPE, and cleaning procedures), is based on practical exercises. Effectiveness is determined through a pre-post comparison, considering patient satisfaction. Category 4, “Multimodal approaches and training programs,” includes five studies (34–38). All are based on addressing several relevant aspects of infection control for cleaning staff, as well as on several individual interventions. Relevant focus areas include aspects of occupational safety (34), ergonomics (34), and aspects of infection prevention, among others. Nosocomial infections (36, 37), transmission routes, hand hygiene (34–38), personal hygiene (35), use of personal protective equipment (34–38), waste management (36), cleaning measures of frequently touched hand, and skin contact surfaces (36, 37). Effectiveness is assessed by before-and-after comparisons and, in some cases, by examining the cleaning status of surfaces using ATP measurement (37).

## 4 Discussion

This scoping review provides a systematic overview of the available evidence on KAP, as well as facilitating and inhibiting factors for IPC in general and hand hygiene in particular among healthcare cleaning staff. Furthermore, interventions aimed at improving hand hygiene behavior and infection prevention practices in this target group were identified. The results show that the current state of research is fragmented, heterogeneous, and predominantly based on descriptive study designs. The identified facilitating and inhibiting factors in the context of IPC and hand hygiene can be clearly attributed to individual, organizational, and societal levels. Intervention approaches specifically tailored to cleaning staff, considering their needs and working conditions, are limited as of now. Interventions focusing exclusively on optimizing hand hygiene for this occupational group are even rarer (5, 6).

The descriptive approach utilized in most studies allows for the systematic assessment of KAP, and context-specific enabling and inhibiting factors within the working environment of the target group. Therefore, the approach can be carried out with comparatively low personnel, material, time, and financial resources. They are particularly valuable for needs assessments, which form an essential basis for developing targeted interventions. However, while descriptive studies can identify associations, they do not allow causal inferences and are prone to various sources of bias, including observer bias, interview bias, recall bias, social desirability bias, and the Hawthorne effect, often resulting in low internal and external validity. Moreover, depending on the specific design, they frequently provide only cross-sectional glimpses, limiting generalizability, especially when sample sizes are small, non-representative, or context specific. Consequently, a substantial proportion of the included studies offers only limited explanatory power, underscoring the need for further research, particularly through qualitative approaches and rigorously designed intervention studies (26).

The included studies exhibit heterogeneity in both their designs and data collection methods. The resulting methodological challenges complicate the comparability of results and the assessment of the strength of evidence, as the validity of the findings depends on the study design. Furthermore, differences in measurement instruments and data collection procedures pose additional challenges in synthesizing and interpreting the findings, and may also lead to inconsistent definitions and evaluation criteria.

Regarding the sample sizes of both descriptive (*n*_*d*_ = 7–1,176) (27, 40) and interventional studies (*n*_*i*_ = 50–807) (32, 37) on the target group of cleaning staff, considerable variability can be observed, which limits the comparability of the studies (20, 27). The diverging sample sizes are particularly relevant with regard to statistical power, the precision of effect estimates, and the external validity of results. This heterogeneity reduces the robustness and interpretability of the available evidence (20, 24, 27). The issue is especially pertinent for intervention studies, where heterogeneous sample sizes complicate the comparison of reported intervention effects. None of the nine included intervention studies reported a formal sample size calculation based on statistical power. Instead, sample sizes were determined by the number of cleaning staff available in the participating hospitals (5, 6, 32–38). The interventions included also have different implementation periods, ranging from 1 month to 1 year. This variability can affect the effectiveness of the measures and further complicates a systematic comparison of the interventions. Other factors that also influence the quality and comparability of intervention studies, such as different study designs, no blinding, often no control groups, and often no follow-up period, are also present (5, 6, 32–38). Consequently, the reported intervention effects should be interpreted with caution, as robust conclusions regarding efficacy are only possible to a limited extent.

Much of the literature reviewed focuses primarily on IPC in general. This is particularly true for the findings, including those concerning promoting and inhibiting factors, KAP, as well as recommended and implemented interventions, which do not specifically address hand hygiene among cleaning staff. This limits the validity of the available evidence, particularly regarding the generalizability and transferability of the results, and consequently the derivation of differentiated, target-group-specific recommendations. At the same time, this indicates a research gap, as target-group-specific determinants and interventions for improving hand hygiene among cleaning staff are currently limited and have rarely been investigated systematically (5).

Across all included sources focusing solely on cleaning staff, a significant proportion of the evidence regarding infection control, particularly hand hygiene, originates predominantly from low- to middle-income countries. Research from high-income countries on cleaning staff is comparatively rare, and this applies to the included guidelines, descriptive studies, and interventional studies alike. Researchers primarily focus on cleaning staff in countries with limited resources, pronounced structural IPC problems, significant threats of nosocomial infections and a higher need for research. There are several possible reasons for the small number of studies from high-income countries. Most hand hygiene measures seem to focus on healthcare professionals in direct contact with patients. Furthermore, particularly in some high-income country settings, outsourcing arrangements, the use of external service providers, and differing organizational responsibilities might complicate researchers' access to cleaning staff and consequently reduce opportunities for research involving this occupational group. Another possible explanation might be that healthcare facilities in high-income countries often adhere to national and international standards, which may blur the need for research and interventions. A focus on other research priorities in HICs, such as the development of novel medical, diagnostic, and technological approaches, could also provide a possible explanation. Furthermore, there is also the possibility of publication bias. Despite the inclusion of gray literature, it is possible that local, institution-specific or non-indexed data collections and reports exist that were not found during the search. At the same time, it is possible that intervention studies with proven efficacy are more likely to be published and indexed, which could also explain the low number of identified studies from HICs.

Regarding the hand hygiene aspects mentioned in the included data sources, a connection can be established between the level of detail of these hand hygiene aspects and the consideration of cleaning staff as an independent occupational group with a differentiated range of activities. Sources explicitly addressing cleaning staff formulate detailed, target-group-oriented hand hygiene moments reflecting the realities of work of this occupational group (5, 6). The guidelines for cleaning staff, particularly the WHO intervention package, include detailed, target-group-, and workplace-specific hand hygiene moments for cleaning staff, which are also incorporated into comprehensive cleaning schedules. The descriptive studies, overall, demonstrate a wide variety of different, activity-oriented hand hygiene moments; however, none of the included studies covers the total of the identified moments. This fragmented and incomplete representation of target group-specific hand hygiene moments demonstrates that while knowledge exists, comprehensive approaches and recommendations might not yet be sufficiently established in the daily work of cleaning staff.

Intervention studies focusing on target group-oriented hand hygiene moments occupy an intermediate position. They define task-related moments but categorize them and present them in a more condensed and less detailed manner. Guidelines and studies on mixed target groups are based on the WHO's classic “Five Moments of Hand Hygiene” (7, 44, 45). This leads to general recommendations primarily geared toward direct patient care activities and therefore not fully encompassing the range of tasks performed by cleaning staff (5). This is a critical issue, as transferring these hand hygiene moments to individual work processes requires extensive expertise in IPC. A lack of specificity can have implications for practical applicability, HHC, and the effectiveness of measures, highlighting the need for target group- and context-sensitive moments in hand hygiene (5, 6).

A large proportion of the descriptive studies included fail to adequately differentiate between hygienic, often alcohol-based, hand disinfection, and handwashing. In those studies that do differentiate, the focus is frequently on handwashing. This contradicts current research and existing recommendations, which, for visibly contaminated hands, emphasize the priority of hygienic hand disinfection over handwashing, due in part to a broader spectrum of activity (especially against non-enveloped viruses) and less skin irritation (1, 2, 12). One potential reason for the increased emphasis on handwashing could be partly due to context-specific factors such as culturally ingrained preferences and structural limitations, e.g., the limited availability of hand sanitizers. This is particularly relevant in low-income countries. Beyond the identification of individual determinants, the interaction between facilitating and inhibiting factors remains insufficiently understood. The identified facilitating and inhibiting factors can be assigned to different levels, e.g., individual, interpersonal, organizational, and societal, and thematic categories, e.g., material resources, education and training, working conditions, recognition, and sociodemographic factors. The available evidence, however, is not yet sufficient to support the development of a robust conceptual framework explaining their interrelationships. Most included studies examined individual determinants in isolation, whereas potential interactions between factors received little attention. In addition, a substantial proportion of the identified determinants originates from studies focusing on IPC more broadly rather than on hand hygiene specifically. Consequently, the evidence regarding relationships between hand hygiene-related determinants remains limited. Given that hand hygiene is embedded within institutional structures and routine work processes, interactions between determinants across different levels and categories appear plausible. This may be particularly relevant for factors associated with education and training. Cleaning staff often constitute a heterogeneous occupational group, characterized by diverse educational backgrounds, varying levels of knowledge regarding infection prevention and hand hygiene, and differing language proficiencies. In this context, language barriers, limited formal education, and insufficient consideration of factors relevant to this specific target group might interact with organizational and workplace-related factors, thereby influencing HHC. These considerations highlight the complexity of the subject and underscore the limitations of approaches that focus solely on individual determinants. Future research should therefore focus not only on identifying relevant determinants but also on investigating their interdependencies and potential mechanisms of influence. Based on a stronger empirical foundation, a multi-layered conceptual framework could be developed to illustrate these relationships and reflect the complexity of hand hygiene behavior among cleaning staff more accurately. In the long term, such a framework may support the design of more targeted, context-sensitive, and practice-oriented interventions.

Both the majority of the identified studies on interventions and all the guidelines included are multifactorial in their focus. These holistic approaches promote a basic understanding of infection control, have greater consideration for existing workflows, and enable synergistic effects between individual aspects, such as the use of PPE, hand hygiene measures, and cleaning procedures. At the same time, they might also overwhelm the target group, especially if they have little prior knowledge or training. Reduced capacity to absorb information and information overload are potential consequences. Multimodal intervention programs potentially also carry the risk of critical individual aspects, such as hand hygiene, not receiving adequate attention due to their broad scope and insufficient implementation due to incommensurate focus. The necessary personnel, time, material, and financial resources may be higher compared to single-pronged interventions. Furthermore, the multifactorial structure of the intervention programs makes it difficult to assess effect sizes of individual components in isolation. Interventions in the form of target group-specific hand hygiene training (5, 6) as well as multimodal approaches and training programs (34–38) appear promising for increasing HHC among cleaning staff. Adapting hand hygiene interventions and training content to the specific workflows and responsibilities of cleaning personnel increases contextual relevance and promotes behavioral changes. Furthermore, these interventions generally require comparatively few personnel, material, time, and financial resources, which increases their feasibility and scalability, particularly in resource-constrained settings (5, 6). Multimodal approaches and training programs are often holistic and promote a comprehensive understanding of infection prevention, considering existing workflows and leveraging synergies between individual components, such as the use of PPE, hand hygiene, and cleaning procedures (34–38). Moreover, interventions should take into account the diverse backgrounds of cleaning staff. Differences regarding prior knowledge of IPC, and specifically HHC, as well as educational levels, literacy skills, and language proficiency should be addressed during the design of these training programs. Knowledge transfer can be facilitated through practical demonstrations, visual learning materials, workplace-based learning approaches, and the use of simple, understandable language, thereby improving the implementation of hand hygiene measures in daily work routines. These considerations also align with the recommendations set forth in the included WHO guidelines (11–14). A combination of both intervention approaches, i.e., target group-specific hand hygiene training and multimodal approaches, could therefore represent a meaningful approach for future intervention development. However, due to the pronounced heterogeneity regarding study designs, outcome measures, statistical analysis methods, interventions, and the limited number of studies investigating the different intervention approaches, this suggestion should be interpreted with caution. Previous systematic reviews on hand hygiene in other healthcare professions have reached similar conclusions. These reviews also revealed significant heterogeneity in the identified sources, which hampered the ability to draw conclusions regarding the effectiveness of clinically relevant improvements in HHC and the reduction of infection and colonization rates (7, 44, 45). Therefore, the planning of future intervention studies should focus on higher study quality to improve methodological rigor and comparability. This applies to studies on this topic and setting across all relevant professions.

Several limitations of this scoping review must be acknowledged, particularly regarding its design and methodology. Suitable sources were identified through a single search of the relevant scientific databases; therefore, publications released after May 16, 2025, were not considered. Due to limited human resources, only 25% of the full texts were reviewed by two reviewers as part of the full-text screening process. This approach carries an increased risk of selection bias and misclassification, while also increasing the scope for interpretation when deciding on suitability. Although the two reviewers showed a high level of agreement when reviewing the titles and abstracts (95.9% / κ = 0.78), indicating consistent application of the predefined inclusion and exclusion criteria, the risk of bias cannot be completely ruled out. Only translation tools were used in the translation process, and no native speakers were consulted for verification. This means that bias may occur as a result of the translation algorithms. It should be noted that this is only relevant for one of the final sources included (6). The methodological and format-related diversity of the included evidence makes it difficult to do equal justice to the different publication types and their respective specifics (39). This heterogeneity was addressed as best as possible by structuring the results into subchapters, separate tables, and, in some cases, graphic visualizations regarding the research questions.

This scoping review also has several strengths: it was conducted according to the PRISMA-ScR guidelines, thus following a structured and methodologically transparent approach that meets current scientific standards (8, 9). By focusing on cleaning staff as the sole target group and excluding sources relating to mixed population groups, the review can also ensure greater target group specificity and consistency. Although the number of included studies was significantly reduced as a result of this adjustment, no substantial changes to the review's main findings were observed. The excluded studies contained only limited specific information regarding the target group of cleaning staff; typically, the results focused on mixed occupational groups without providing separate analyses for cleaners. Consequently, their exclusion did not alter the overall conclusions. Furthermore, no language restrictions were imposed during the search and screening process, enabling the inclusion of a broad spectrum of international literature (10). To the authors' current knowledge, this scoping review represents the first comprehensive and systematic overview of hand hygiene among cleaning staff in healthcare settings. The study design of a scoping review allows for the exploration of a previously under-researched topic, taking into account the specific target group. In addition, various publication formats, including guidelines, descriptive and interventional full-text studies, and abstracts without available full texts, are taken into consideration (8, 9). It also incorporated diverse thematic focuses within hand hygiene, including facilitating and inhibiting factors, KAP, as well as recommended and already implemented intervention approaches. Furthermore, the scoping review enabled the systematic identification of substantial gaps in the evidence that limit the validity of the current state of research (8, 9). These findings form the basis for the implications and recommendations for future research projects presented below.

As demonstrated in this review, there remains a need for methodologically diverse research that systematically classifies, deepens, and expands upon existing knowledge. This includes both descriptive studies examining the current situation and studies on the development and evaluation of target group-specific interventions. Due to the current, highly heterogeneous quality of studies, greater attention should be paid to ensuring higher study quality during planning in order to enable the use of existing critical appraisal tools when conducting systematic reviews and meta-analyses. Future research projects should address the limited geographical scope of currently available research. Studies on the implementation and effectiveness of interventions are currently required from countries of all income levels. To increase the methodological diversity and significance of the collected data, the integration of microbiological testing methods, particularly of anonymized samples from employees' hands and, if necessary, also from gloves and surfaces, can be considered (17). This applies to both descriptive studies on the status quo and the evaluation of intervention effects. For example, contact plate and swab tests are suitable, as they are simple and cost-effective to perform in practice. In addition to determining the total bacterial count (bacteria, yeasts, and molds) on the sampled surface, selective testing methods for the detection of specific microorganisms, such as relevant pathogens of nosocomial infections, can also be integrated. In both descriptive and interventional studies, future research should focus more on a differentiated analysis of handwashing, hand disinfection, and the pathogen- and situation-dependent combination of both hand hygiene methods. If possible, the priority of hand disinfection over handwashing should be emphasized (1, 2, 12).

With regard to descriptive studies, it should be noted that cross-sectional studies based on one-off data collections only represent glimpse. Future research should increasingly utilize longitudinal study designs examining the target group over a longer period and at multiple measurement points, thus revealing changes in behavior and the surrounding circumstances (25, 31). Furthermore, future research projects should focus more intently on specific aspects such as hand hygiene, thereby enabling a more in-depth investigation. This would allow the investigation of influencing factors for which there currently is no information regarding hand hygiene among the target group of cleaning staff specifically. Future intervention studies should focus more strongly on individual aspects of IPC, particularly hand hygiene, so as to consider specific, context-sensitive, and target-group-appropriate mechanisms of action (5, 6, 22, 32–36, 38). At the same time, hand hygiene interventions should not be implemented in isolation but rather integrated into existing workflows and cleaning processes (5, 6). Future intervention-related research projects should also give greater consideration to the social, cultural, and linguistic characteristics of the target group, for example, by integrating vocational training and low-threshold, language-sensitive training approaches (2, 11, 12).

## 5 Conclusion

This scoping review highlights the central, yet under-researched role of hand hygiene in infection prevention among cleaning staff in the healthcare sector. Current available evidence regarding KAP, as well as factors that promote or hinder hand hygiene, is fragmented, thematically and methodologically heterogeneous, and predominantly descriptive. The findings further emphasize the need for internationally recognized and target group-specific guidelines that account for the working conditions of cleaning staff and clearly define relevant hand hygiene practices. Future research should focus on the development and evaluation of context-sensitive interventions to support sustainable improvements in hand hygiene among cleaning staff.
