# Supplementary material for: Hand hygiene among cleaning staff in acute care hospitals: a scoping review
Source: Front Public Health. 2026 Jul 16;14:1800229. doi: 10.3389/fpubh.2026.1800229 (PMC13422454; doi:10.3389/fpubh.2026.1800229)
Supplement: Supplementary file 1 [file Datasheet_1.pdf]

Appendix - Appendix 1

Table 1 (Appendix 1): Detailed table of inclusion and exclusion criteria based on the PCC scheme:

Inclusion criteria	Exclusion criteria
Population (P)	
- Cleaning staff in somatic acute care hospitals, worldwide,<br>- Occupational groups without direct patient contact in somatic acute care hospitals (e.g. cleaners, housekeeping staff, ward assistants, house technicians, etc.), worldwide,	- Cleaning staff employed outside of somatic acute care hospitals, e.g. psychiatric facilities, office, glass, factory, and transport cleaning,<br>- Ward assistants, house technicians employed outside somatic acute care hospitals,<br>- Mixed populations (i.e. occupational groups without and with direct patient contact, e.g. healthcare professionals), worldwide.<br>- Only occupational groups with direct patient contact, e.g. nurses, physicians.
Concept (C)	
- Interventions: No interventions, e.g. in observational studies that record the current situation at compliance and adherence rates, behaviors, attitudes, promoting and inhibiting factors of hand hygiene, approaches based on psycho-social models, context-based approaches/adaptations in the setting, visual and written reminders, nudging approach (reminders, pictograms, emojis), observation and feedback, education and training measures (programs, videos, etc.), electronic systems, e.g. on dispenser systems / electronic monitoring systems (cf. v. Lengerke et al., 2017, p. 1 ff.; Fabrizio et al., 2022, p. 1900 f.), multi-factorial interventions which, in addition to focusing on hand hygiene, also include aspects such as general knowledge of hospital cleaning and surface cleaning in hygiene-critical areas (e.g. cleaning and disinfection of surfaces, etc.), general knowledge of hygiene and infection prevention, use of personal protective equipment, skin care and skin protection (use of skin care and protective creams and protective gloves).	- Interventions: No interventions, e.g. in observational studies that record the status about surface hygiene measures, measures that focus on skin protection and skin care without taking hygiene-related aspects into account (e.g. use of skin protection and skin care creams and protective gloves), measures and interventions that only deal with the safe disposal of healthcare waste, without reference to hand hygiene, measures and interventions that only deal with the safe disposal of linen in healthcare settings, with no reference to hand hygiene, measures and interventions that focus solely on the prevention of predominantly airborne infections, such as COVID-19, influenza, tuberculosis and other colds, without reference to hand hygiene, measures and interventions that only address the topic of immunization, i.e. attitudes towards immunization or antibody status, e.g. for rubella, measles, mumps, varicella-zoster viruses, etc., without reference to hand hygiene,
Context (C)	
- Acute care hospitals, worldwide.	- Veterinary medicine facilities,<br>- Facilities such as psychiatric facilities, office, glass, factory, and transport cleaning.
Types of Sources	
- Study design: experimental and quasi-experimental study designs including randomized controlled trials, non-randomized controlled trials, before and after studies, interrupted time-series studies, analytical observational studies including prospective and retrospective cohort studies, case-control studies, analytical cross-sectional studies, descriptive observational study designs including case series, individual case reports, descriptive cross-sectional studies, systematic reviews with and without meta-analysis, scoping reviews.<br>- Additional literature: expert surveys, reports from professional societies, guidelines.	

Appendix 2

Appendix 2.1 – Search strings systematic literature research, databases

1. Population: (("cleaner*" OR "cleaning staff" OR "cleaning worker*" OR "housekeep*" OR "facility manage*" OR "janitor*" OR "custodia*" OR "sanitary work*" OR "housekeeping, hospital")

2. Concept: ("infection control*" OR "infection prevention" OR "hand hygien*" OR "hand disinfect*" OR "hand sanitiz*" OR "hand sanitis*" OR "hand clean*" OR "hand wash*" OR "handwashing*" OR "hand hygiene adherence" OR "hand hygiene compliance" OR ("Infection Control" OR "Hand Hygiene"))

3. Context: ("hospital*" OR "clinic*" OR "healthcare facilit*" OR "Hospitals"))

Appendix 2.2 – Search strings systematic literature research, databases

Table 2 (Appendix 2): Search string systematic literature search for replication of the search, database search, MEDLINE (via PubMed®), based on the PCC scheme

Population		Concept		Context		Period
(("cleaner*"[All Fields] OR "cleaning staff"[All Fields] OR "cleaning worker*"[All Fields] OR "housekeep*"[All Fields] OR "facility manage*"[All Fields] OR "janitor*"[All Fields] OR "custodia*"[All Fields] OR "sanitary work*"[All Fields] OR "housekeeping, hospital"[MeSH Terms])	AND	("infection control*"[All Fields] OR "infection prevention"[All Fields] OR "hand hygien*"[All Fields] OR "hand disinfect*"[All Fields] OR "hand sanitiz*"[All Fields] OR "hand sanitis*"[All Fields] OR "hand clean*"[All Fields] OR "hand wash*"[All Fields] OR "handwashing*"[All Fields] OR "hand hygiene adherence"[All Fields] OR "hand hygiene compliance"[All Fields] OR ("Infection Control"[MeSH Terms] OR "Hand Hygiene"[MeSH Terms]))	AND	("hospital*"[All Fields] OR "clinic*"[All Fields] OR "healthcare facilit*"[All Fields] OR "Hospitals"[MeSH Terms]))	AND	(2009:2025[pdat])

Table 3 (Appendix 2): Search string systematic literature search for replication of the search, database search, Cochrane Library, based on the PCC scheme

Population		Concept		Context		Period
(cleaner* OR "cleaning staff" OR ("cleaning" NEXT worker*) OR housekeep* OR ("facility" NEXT manage*) OR janitor* OR custodia* OR ("sanitary" NEXT work*) OR [mh "housekeeping, hospital"])	AND	(("infection" NEXT control*) OR "infection prevention" OR ("hand" NEXT hygien*) OR ("hand" NEXT disinfect*) OR ("hand" NEXT sanitiz*) OR ("hand" NEXT sanitis*) OR ("hand" NEXT clean*) OR ("hand" NEXT wash*) OR handwashing* OR "hand hygiene adherence" OR	AND	(hospital* OR clinic* OR ("healthcare" NEXT facilit*) OR [mh Hospitals])	AND	(2009-2025)

		"hand hygiene compliance" OR [mh "Infection Control"])				

Table 4 (Appendix 2): Search string systematic literature search for replication of the search, database search, Embase, based on the PCC scheme

Population		Concept		Context		Period
(cleaner* OR 'cleaning staff' OR 'cleaning worker*' OR housekeep* OR 'facility manage*' OR janitor* OR custodia* OR 'sanitary work*'OR 'cleaning staff'/exp OR 'hospital service'/exp)	AND	('infection control*' OR 'infection prevention' OR 'hand hygien*' OR 'hand disinfect*' OR 'hand sanitiz*' OR 'hand sanitis*' OR 'hand clean*' OR 'hand wash*' OR handwashing* OR 'hand hygiene adherence' OR 'hand hygiene compliance' OR 'infection control'/exp OR 'hand washing'/exp)	AND	(hospital* OR clinic* OR 'healthcare facilit*' OR 'hospital'/exp)	AND	(2009-2025)

Table 5 (Appendix 2): Search string systematic literature search for replication of the search, database search, CINAHL Database (via EBSCO), based on the PCC scheme

Population		Concept		Context		Period
(cleaner* OR "cleaning staff" OR "cleaning worker*" OR housekeep* OR "facility manage*" OR janitor* OR custodia* OR "sanitary work*" OR 'MM "Housekeeping Department+")	AND	('"infection control*" OR "infection prevention" OR "hand hygien*" OR "hand disinfect*" OR "hand sanitiz*" OR "hand sanitis*" OR "hand clean*" OR "hand wash*" OR handwashing* OR "hand hygiene adherence" OR "hand hygiene compliance" OR 'MM "Infection Control+" OR MM "Handwashing+")	AND	(hospital* OR clinic* OR "healthcare facilit*" OR MH "Hospitals+")	AND	(2009-2025)

Table 6 (Appendix 2): Search string systematic literature search, database search for replication of the search, Scopus, based on the PCC scheme

Population		Concept		Context		Period
ALL(cleaner*) OR ALL("cleaning staff") OR ALL("cleaning worker*") OR ALL(housekeep*) OR ALL("facility manage*") OR ALL(janitor*) OR ALL(custodia*) OR ALL("sanitary work*")	AND	'ALL("infection control*") OR ALL("infection prevention") OR ALL("hand hygien*") OR ALL("hand disinfect*") OR ALL("hand sanitiz*") OR ALL("hand sanitis*") OR ALL("hand clean*") OR ALL("hand wash*") OR ALL(handwashing*) OR "hand hygiene adherence" OR "hand hygiene compliance"	AND	ALL(hospital*) OR ALL(clinic*) OR ALL("healthcare facilit*")	AND	(2009-2025)

Appendix 2.3 - Search for grey literature - search strings:

Table 7 (Appendix 2): Search string systematic literature search for replication of the search, search for grey literature

Institution, database	Keywords used
World Health Organization (WHO), Institutional Repository for Information Sharing (IRIS)	Handhygiene
	Cleaning staff
	Cleaning worker
	"cleaning staff" OR "cleaning worker*"
Bielefeld University, Bielefeld Academic Search Engine (BASE)	Cleaning staff (Advanced search; All fields; 2009-2025; Filter disabled: Prefer open access documents)
	cleaning staff AND hand hygiene (Advanced search; All fields; 2009-2025; Filter disabled: Prefer open access documents)
U.S. government, Science.gov	"cleaning staff"
	"cleaning staff" AND handhygiene
	cleaning staff and hand hygiene
Brassey, J, Price, C., TRIP Medical Database	"cleaning staff" (2009-2025)
	"cleaning staff" AND handhygiene (2009 - 2025)
	"cleaning staff" AND handhygiene (2009 - 2025)

Appendix 2.4 - Use of Consensus as a supplementary hand search

Table 8 (Appendix 2): Consensus, questions used to identify relevant studies

Questions used
What do we know about the hand hygiene behavior of cleaning staff in hospitals?
What attitudes do hospital cleaning staff have towards hand hygiene?
What inhibiting and promoting factors are known to influence the hand hygiene behavior of cleaning staff in hospitals? Which barriers are known?
What measures can be implemented in the long term to improve the hand hygiene behavior of cleaning staff in hospitals?

Appendix 3:

Appendix 3.1 – Data extraction, three categories

Category I – Guidelines: database, publishing institution, country, document type, concept, thematic focus, type of intervention, relevant text section.

Category II – Full-text studies: database, authors (year of publication), country, setting, study population, sample size (n), document type, study design, methodology, thematic focus and relevant keywords, type of intervention, results, key findings, conclusion, and recommendations.

Category III – Conference Abstracts/ Poster Abstracts without accompanying full text: database, authors (year of publication), country, setting, study population, sample size (n), document type, study design, methodology, thematic focus and relevant keywords, type of intervention, results, key findings, conclusion, and recommendations.

Appedix 3.2 – Part 1 and 2: Extraction table excerpt (Excel), full-text studies, sample

Figure 1 (Appendix 3): Part 1 of the extraction table

No.	Database	Authors (year of publication)	Country	Setting, study population, sample size (n) (if applicable)	Document type	Study design/ Study category (observational study, intervention study)	Methodology	Thematic focus and relevant Keywords
7	Scopus, Embase	Ahmed, I.; Farooq, U.; Rabia, M. et al. (2011)	Asia, Pakistan (Rawalpindi district)	Tertiary care hospital (600 beds), hospital sanitary worker (n = 88, female: nf = 46, 52,28 %; male: nm = 42, 47,72 %) (Full time sanitary workers)	Study	Cross sectional study (Interview)	All sanitary workers who have been working in the hopital for more than three months were interviewed by a doctor in a language they understand. Interviews were conducted on the basis of an anonymous, self-explanatory, comprehensive questionnaire.Yes/no questions or questions about time periods.	Assessment of awareness and knowledge among sanitary workers in hospitals with regard to infection control (including training interventions, vaccinations, hand hygiene, personal protective equipment (PPE), handling of infectious waste). Relevant Keywords (by the authors): Sanitary workers, waste disposal, hospital wastes

Figure 2 (Appendix 3): Part 2 of the extraction table

Type of intervention, comparative alternative if applicable, incl. details (if applicable)	Outcomes, including details (e.g. how they are measured) (if applicable)	Main results	Conclusion and Recommendations (by the authors)
No intervention, recording of the current situation.	Questions on topics such as medical examinations and consultations before/after starting work, vaccinations, availability and use of personal protective equipment (PPE) (including protective gloves and masks), handling of infectious waste, use and handling of cleaning agents and disinfectants, hand hygiene behaviour, awareness of infectious diseases and infection prevention measures.	None of the sanitation workers had a medical examination before or during work (100%, n = 88).<br>4.54% (3.46%-5.25%) had received training before starting work and none during work.<br>23.86% (n = 21) used protective gloves, while 76.14% (n = 67) did not. Surgical gloves were mostly used.<br>Hand hygiene measures after handling/contacting medical waste:<br>Handwashing with water: yes (39.77%, n = 35), no (60.23%, n = 53);<br>Handwashing with soap and water: yes (52.27%, n = 46), no (47.73%, n = 42);<br>Use of antiseptics: yes (7.95%, n = 7), no (97.05%, n = 81).<br>Knowledge and awareness of infectious diseases: yes (27.27%, n = 24), no (72.73%, n = 64).<br>Basic personal hygiene among sanitary workers is also important to reduce the risk of infection posed by biomedical waste.	Education and training of sanitary workers is recommended. This can reduce the spread of infectious diseases and improve the health of the cleaning staff and their efficiency.
[illegible]	[illegible]	[illegible]	[illegible]
